# Appraising the role of circulating concentrations of micro-nutrients in epithelial ovarian cancer risk: A Mendelian randomization analysis

**DOI:** 10.1038/s41598-020-63909-5

**Published:** 2020-04-30

**Authors:** Yan Guo, Yunlong Lu, Hongchuan Jin

**Affiliations:** 1 School of Medicine, Zhejiang University, Hangzhou, 310058 China; 2 Laboratory of Cancer Biology, Key Lab of Biotherapy in Zhejiang, Sir Run Run Shaw Hospital, Medical School of Zhejiang University, Hangzhou, 310000 China

**Keywords:** Epidemiology, Genetics research

## Abstract

To determine the causality of micro-nutrients concentrations and risk of ovarian cancer using the Mendelian randomization approach. Analyses were conducted using summary statistics data for SNPs robustly associated with concentrations of thirteen micro-nutrients (iron, copper, zinc, calcium, magnesium, phosphorus, selenium, vitamin A, β-carotene, vitamin B6, vitamin B12, vitamin E, folate). The corresponding data for ovarian cancer were obtained from the Ovarian Cancer Association Consortium (25,509 cases and 40,941 controls). In standard Mendelian randomization analysis, the odds ratios (OR) of invasive epithelial ovarian cancer were 0.14 (95% CI, 0.03–0.70; P = 0.02) per 0.1 mmol/L (about one standard deviation, SD) increase in genetically predicted magnesium concentration, 1.04 (95% CI, 1.00–1.09; P = 0.03) per 0.3 μmol/liter (about one SD) increase in genetically predicted β-carotene concentration. The OR of low malignant potential tumours were 0.82 (95% CI, 0.76–0.90; P = 1.01 × 10^−5^) per 0.3 μmol/liter (about one SD) increase in β-carotene concentration, 1.42 (95% CI, 1.21–1.68; P = 3 × 10^−5^) per 153 pmol/L (about one SD) increase in vitamin B12 concentration, 0.21 (95% CI, 0.06–0.76; P = 0.02) per 6 mg/L (about one SD) increase in vitamin E concentration. No significant associations of other micro-nutrients and ovarian cancer were observed. This study found that an increased risk of invasive epithelial ovarian cancer was observed with a genetically higher concentration of β-carotene, whereas a decreased risk of invasive epithelial ovarian cancer was found with a higher concentration of magnesium. As for low malignant potential tumours, increased concentration of vitamin B12 could increase the risk of low malignant potential tumours, while increased concentrations of β-carotene and vitamin E could lower the risk of low malignant potential tumours.

## Introduction

Globally, around 300,000 new cases and 185,000 deaths occur each year, making ovarian cancer the eighth most common cause of death in female cancer, and the second most common cause of death in gynecological cancer (after cervical cancer)^1^. The prognosis of ovarian cancer is generally poor, with a 5-year survival rate of only 48% after diagnosis. In contrast, the 5-year survival rate of breast cancer is 90%^2^. Despite advances in modern medicine, the survival rate of ovarian cancer has changed little over the decades, even in the resource-rich countries such as the United States and Canada^2,3^. These frustrating figures are in part due to the lack of effective screening tests for early detection of ovarian cancer and the lack of early, specific symptoms that result in diagnostic delays^2^. Given the limited success of secondary prevention strategies and high cure rate of early-stage disease, at present, the best opportunity for disease control and even cure might be at primary prevention. Stratified analyses across clinically distinct histotypes are necessary for prevention and treatment of ovarian cancer. Ovarian epithelial tumours can be classified according to the following histological subtypes: serous, mucinous, endometrioid, clear cell, transitional cell, Brenner, small cell, mixed mesodermal and undifferentiated. Usually each subtype can be classified as benign, borderline (low malignant potential, LMP) and malignant (invasive), in which the prognosis of invasive epithelial ovarian cancer is generally poorer^4^.

Micronutrients include vitamins and minerals required in very small quantities in our bodies. They’re critical for a number of important functions, including growth, development and disease prevention^5^. However, the role of nutrients in the development of ovarian cancer remains unclear. Firstly because the literature on the circulating concentrations of minerals and vitamins with risk of ovarian cancer is generally limited, except vitamin D^6,7^. Previous research of the cellular mechanism of vitamin D in ovarian cancer suggested that vitamin D played a critical role in antitumorigenic activities by regulating cellular proliferation and metabolism through genomic and nongenomic signal transduction pathways^6^. However, the role of other micronutrients in ovarian cancer and underlying mechanisms need to be unraveled. Besides, there has been little agreement in the published literature on the role of micro-nutrients in ovarian cancer. For instance, the California Teachers Study prospective cohort study showed that higher intake of β-carotene was associated with a 41% higher risk for ovarian cancer^8^, but another population-based case-control retrospective study suggested that the serum concentration of β-carotene was inversely correlated with the risk of ovarian cancer^9^. Furthermore, given the observational design of most available studies on micro-nutrients and ovarian cancer risk, it is uncertain whether the observed associations are causal and independent of other confounding factors.

Mendelian randomization (MR) is a genetic epidemiological approach that exploits germline genetic variants as unbiased proxies for exposure of interest to infer causality^10–12^. Since germline genetic variants are randomly assorted at meiosis, MR analyses should be less likely to be confounded by environmental factors than conventional observational studies. Additionally, since at the time of conception, germline genetic variants are set and cannot be changed by subsequent processes of disease, MR analyzes are not influenced by reverse causality bias. Another benefit of MR is that it can be applied using summary genetic association data from two independent samples (known as the 2-sample MR approach) representing (1) associations of genetic variant-risk factor and (2) associations of genetic variant-outcome. This approach provides an effective and statistically robust method for assessing the causal relationships between risk factors and outcomes. However, to our knowledge, the effects of micronutrient in ovarian cancer have not been evaluated on a large scale with the use of MR.

The aim of our study is to investigate whether micro-nutrients concentrations are causally associated with epithelial ovarian cancer as a whole or any of its histotypes by applying the 2-sample MR approach.

## Results

For each risk factor, the number of SNPs included in the genetic instruments was provided in Table 1. Complete Mendelian randomization analyses were presented in Tables S1 and S2 for invasive epithelial ovarian cancer histotypes and low malignant potential tumours. Scatter plots for findings showing strong or suggestive evidence of association in standard IVW analyses were presented in S1 Plots. Leave-one-out plots were presented in S2 Plots.

**Table 1:** Catalogue of Dataset used for genetic instruments.

Exposures	GWAS	Race	Number of SNPs available^a^	Number of SNPs used^b^	% of variance explained	Sample Size	F-statistic
Iron (Fe)	Benyamin *et al*.^43^	Europeans	5	3^†^	3.4	48,972	345
Copper (Cu)	Evans *et al*.^44^	Australians	2	2	5	2,603	68
Zinc (Zn)	Evans *et al*.^44^	Australians	3	3	8	2,603	75
Calcium (Ca)	O’Seaghdha *et al*.^45^	Europeans	7	7	0.9	61,054	79
Magnesium (Mg)	Meyer *et al*.^40^	Europeans	5	5	1.6	15,366	50
Phosphorus (P)	Kestenbaum *et al*.^46^	Europeans	4	4	1.5	16,264	62
Selenium (Se)	Evans *et al*.^44^	Australians, British	2	2	4	5,477	114
Vitamin A (Vit A)	Mondul *et al*.^47^	Caucasians	2	2	2.3	8,902	105
β-carotene	Ferrucci *et al*.^48^	Europeans	4	4	4.5	3,918	46
Vitamin B6 (Vit B6)	Tanaka *et al*.^49^	Europeans	1	1	1	1,864	19
Vitamin B12 (Vit B12)	Grarup *et al*.^26^	Europeans	15	14	6.3	45,575	204
Vitamin E (Vit E)	Major *et al*.^50^	Europeans	3	3	1.7	5,006	29
Folate	Grarup *et al*.^26^	Europeans	3	3	1	37,465	126

^a^Corresponds to the number of SNPs available at the genome-wide significance level (P < 5 × 10^−8^).

^b^Corresponds to the number of SNPs (or linkage disequilibrium proxies) available in ovarian cancer datasets.

^†^For serum iron, three out of the five available genome-wide significant SNPs were used, because these SNPs showed a concordant effect on serum iron, ferritin, transferrin and transferrin saturation.

GWAS, genome-wide association study; SNP, single nucleotide polymorphism.

### Causality between minerals and epithelial ovarian cancer

**Figure 1:**
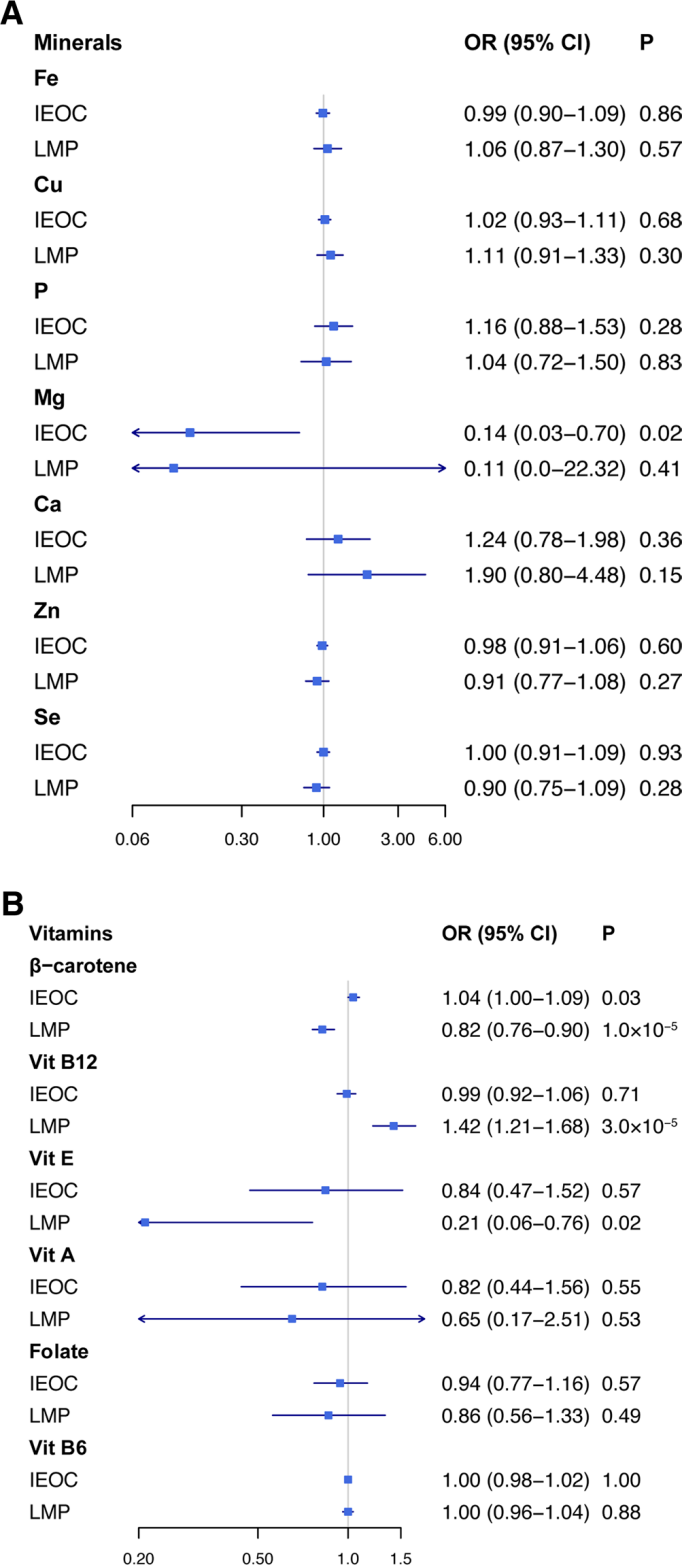
IVW estimates for the association of concentrations of micro-nutrients with risk of epithelial ovarian cancer. IEOC, invasive epithelial ovarian cancer; LMP, low malignant potential epithelial ovarian tumours; CI, confidence interval; OR, odds ratio.

In analyses examining invasive epithelial ovarian cancer and low malignant potential tumours, magnesium was the only mineral that was negatively correlated with invasive epithelial ovarian cancer in the standard IVW analysis, with an OR of 0.14 (95%CI, 0.03–0.70; P = 0.02) (Fig. 1). The correlation was in line with complementary analyses using weighted median method (OR, 0.19; 95% CI, 0.02–1.67) and MR-RAPS (OR, 0.12; 95% CI, 0.02–0.73) (Table S1). No outlier was detected using MR-PRESSO. No indication of directional pleiotropy was found in the MR-Egger analysis (*P* for MR-Egger intercept = 0.26).

In analyses examining ovarian cancer histotypes, calcium and phosphorus were positively correlated with mucinous borderline tumours and high grade serous carcinoma, respectively. The standard IVW estimate showed that the OR of mucinous borderline tumours per SD (0.5 mg/dL) increase in calcium concentration was 3.29 (95% CI, 1.14–9.53; P = 0.03). Similar trends were shown when employing weighted median estimators (OR, 2.71; 95% CI, 0.58–12.71) and MR-RAPS (OR, 3.20; 95% CI, 0.80–12.79), though without significance. No outlier and directional pleiotropy were detected (*P* for MR-Egger intercept = 0.96) (Table S1). The IVW estimate showed that the OR of high grade serous carcinoma per SD (0.5 mg/dL) increase in phosphorus concentration was 1.40 (95% CI, 1.05–1.85; P = 0.02). The corresponding OR was 1.40 (95% CI, 0.81–2.41) in the analysis based on the weighted median method. No outlier and directional pleiotropy were found (*P* for MR-Egger intercept = 0.99) (Table S1).

### Causality between vitamins and epithelial ovarian cancer

Among six vitamins, β-carotene was significantly associated with risk of invasive epithelial ovarian cancer (including histotypes) and low malignant potential tumours. Genetically predicted serum β-carotene levels were positively associated with invasive epithelial ovarian cancer (OR, 1.04; 95% CI, 1.00–1.09; P = 0.03) (Fig. 1), mucinous carcinoma (OR, 1.21; 95% CI, 1.07–1.37; P = 0.003), and endometrioid carcinoma (OR, 1.10; 95% CI, 1.05–1.15; P = 4.02 × 10^−5^) in standard IVW analysis. In contrast, β-carotene was negatively correlated with low grade serous carcinoma (OR, 0.76; 95% CI, 0.67–0.86; P = 1.85 × 10^−5^), low malignant potential tumours (OR, 0.82; 95% CI, 0.76–0.90; P = 1.01 × 10^−5^)(Fig. 1) and mucinous borderline tumours (OR, 0.57; 95% CI, 0.53–0.61; P = 3.89 × 10^−53^). No outlier and directional pleiotropy were detected (Table S2).

In analyses performed for invasive epithelial ovarian cancer and low malignant potential tumours, genetically predicted vitamin E levels were inversely associated with low malignant potential tumours in the standard IVW analysis, with an OR of 0.21 (95% CI, 0.06–0.76; P = 0.02) per 1 SD (6.0 mg/L) increase of serum vitamin E levels (Fig. 1). Genetically predicted vitamin B12 concentration was not significantly associated with low malignant potential tumours in the IVW method (OR, 1.16; 95% CI, 0.96–1.41; P = 0.12) until the MR-PRESSO test detected one outlier (rs12272669). After adjustment for the outlier, there was a suggestive positive association between vitamin B12 and low malignant potential tumours by using the IVW analysis (OR, 1.42; 95% CI, 1.21–1.68; P = 3 × 10^−5^), the weighted median analysis (OR, 1.33; 95% CI, 1.05–1.69; P = 0.02), the MR-Egger regression (OR, 1.57; 95% CI, 1.08–2.28; P = 0.04), and MR-RAPS (OR, 1.41; 95% CI, 1.17–1.70; P = 3 × 10^−4^) (Fig. 1, Table S2). There was no indication of directional pleiotropy (*P* for MR-Egger intercept = 0.57).

In analyses performed for ovarian cancer histotypes, a negative association was observed between genetically determined vitamin B6 concentrations and clear cell carcinoma based on the MR-RAPS approach (OR, 0.91; 95% CI, 0.85–0.97; P = 0.005). Genetically predicted vitamin E levels were inversely associated with serous borderline tumours in the standard IVW analysis (OR, 0.12; 95% CI, 0.02–0.56; P = 0.01), and in the weighted median analysis (OR, 0.12; 95% CI, 0.01–1.06). There was no evidence of directional pleiotropy (*P* for MR-Egger intercept = 0.45) (Table S2).

## Discussion

In this comprehensive Mendelian randomization analysis of thirteen nutrients concentrations with risk of epithelial ovarian cancer, an increased risk of invasive epithelial ovarian cancer was related to a higher concentration of β-carotene, while a decreased risk of invasive epithelial ovarian cancer was related to a higher concentration of magnesium. As for the risk of low malignant potential tumours, it had a positive correlation with the concentration of vitamin B12, and negative correlations with β-carotene and vitamin E. When stratified on histotypes, most risk factors (calcium, phosphorus and vitamin B6) were associated with one or more subtypes, underscoring the heterogeneous nature of this disease.

There is a paucity of literature on the association of circulating magnesium and the risk of epithelial ovarian cancer. Only one case-control study in Taiwan^13^, involving 933 ovarian cancer deaths and 933 deaths from other causes, from 1986 to 2000, reported on the possible association between the risk of ovarian cancer and the levels of magnesium and calcium in drinking water. This study reported that there was a significant protective effect of magnesium intake from water on the risk of ovarian cancer, supporting our findings. A randomized intervention trial showed that the average serum magnesium concentration increased from 0.84 mmol/L to 0.87 mmol/L after 12 weeks supplementation with 350 mg magnesium daily^14^, and the increase of serum magnesium corresponds to about 0.3 SD in our MR study. Low magnesium promoted inflammation which induced genetic instability and might cause mutation in synergy with low magnesium, thus allowing the generation of highly aggressive cells^15^. Inflammation was very intense, and Tumour Necrosis Factor α (TNFα) was increased in the serum of magnesium-deficient mice^15^. TNFα, the target of NF-κB and prototypical pro-inflammatory cytokine, could enhance tumour invasion by facilitating the epithelial to mesenchymal transition, then augment the capacity of cancer cells to metastatize^16^. Further, magnesium was an absolute requirement for the function of *NM23-H1*, a metastasis-suppressor gene. Metastasis was accelerated in *NM23-H1* knockout mice^17^. Therefore, low magnesium availability impaired the anti-metastatic activity of *NM23-H1* in mice. Additionally, senescence of endothelial and fibroblast cells was promoted by low magnesium^18^, and senescent cells could change the tissue environment in a way that synergistically potentiated tumour growth and development with carcinogenic mutations^19^.

The role of β-carotene in ovarian cancer development is unclear. Our result showed that higher concentration of β-carotene could reduce the risk of low malignant potential tumours, which was in line with a population-based study of 549 cases of ovarian cancer and 516 controls, demonstrating that intakes of carotene from food and supplements were significantly and inversely correlated with risk of ovarian cancer, predominantly among postmenopausal women^9^. This result can readily be understood for the supportive function of β-carotene, such as antioxidation, inhibition of tumour initiation and promotion, and enhancement of immunity and cellular maturation^20^. Data from a randomized controlled trial indicated that the average serum concentration of β-carotene increased from 0.47 μmol/liter to 1.14 μmol/liter with 3 mg daily oral supplement of β-carotene for one month^21^, and the increase of serum β-carotene corresponds to about 2 SD in our MR study. However, an increased risk of invasive epithelial ovarian cancer was also observed for genetically higher concentrations of β-carotene in our study. In accordance with this result, the California Teachers’ Study has demonstrated that higher intake of β-carotene was associated with a 41% higher risk for ovarian cancer^8^. Abhishek Goyal^22^ *et al*. evaluated all-cause, cancer and cardiovascular mortality risks associated with quintiles (Q1-Q5) of serum antioxidants, including β-carotene, in 16,008 adult NHANES III (The Third National Health and Nutrition Examination Survey, 1988–1994) participants. The results of this study indicated that cancer mortality risks decreased from Q1-Q2 of β-carotene and did not change significantly with higher levels of β-carotene. Clinical trials of vitamin supplementation with cancer incidence and mortality as outcomes suggested that 12 years of supplementation with β-carotene produced neither benefit nor harm to the incidence of malignant tumours^23^. These results (i.e., the different roles of β-carotene in different histotypes of ovarian cancer) may appear confusing. First, the reliability of results needs to be checked. The reliability of MR results relies on the MR assumptions and pleiotropy occurs when a genetic variant is associated with more than one phenotype. As β-carotene needs to be consumed (i.e., they are not synthesised endogenously), it is possible that genetic variants may influence β-carotene levels indirectly through altered dietary preferences, like vegetables and fruit consumption. In order to minimize the confounding effect of any dietary preferences, we examined the associated phenotype of each genetic instruments, and no correlation between dietary preferences and genetic variants was found^24,25^. Second, the tissue and cell specific effects of circulating β-carotene cannot be ruled out, as well as the unknown difference in genetic or epigenetic alterations between these two histotypes. Therefore, As the first to explore the correlation of β-carotene and risk of ovarian cancer histotypes, our study indicated that further studies, taking cancer histotypes into account, will need to be undertaken.

In analyses examining the correlation between the concentration of vitamin B12 and risk of low malignant potential tumours, one outlier (rs12272669) was detected by MR-PRESSO and leave-one-out analyses (S2 plots). A strong positive correlation between vitamin B12 and low malignant potential tumours was observed after the exclusion of rs12272669 (OR, 1.42; 95% CI, 1.21–1.68; P = 2 × 10^−5^). Rs12272669, located in the *MMACHC* gene, was involved in binding and intracellular trafficking of vitamin B12^26^. The effect allele frequency of rs12272669 was 0.22%, which might give rise to the unstable effects on analysis. To date, epidemiological studies have largely failed to provide evidence to support the association of vitamin B12 with risk of ovarian cancer^27^. However, our finding reported here shed new light on the positive correlation of vitamin B12 and risk of low malignant potential tumours. The precise mechanism of vitamin B12 in ovarian cancer remains to be elucidated.

A randomized intervention study showed that the supplementation of 200 mg/d vitamin E for one month increased the serum vitamin E level from 25.6 μmol/L to 49 μmol/L, which corresponds to about 1.7 SD in our MR study^28^. In our study, a higher concentration of vitamin E was observed to reduce risk of low malignant potential tumours. However, the observed correlation between vitamin E and invasive epithelial ovarian cancer in this study was not significant. Prior studies that have noted the importance of vitamin E in preventing cancer: various cancer-promoting pathways such as COX and 5-LOX-catalyzed eicosanoids could be blocked, key transcription factors such as NF-κB and STAT3 could be inhibited, and cancer cell death could be induced by vitamin E via modulating various signaling pathways, including sphingolipid metabolism^29^. Moreover, those natural forms of vitamin E that were easy to metabolize had stronger anti-inflammatory effects than some unmetabolized vitamers^30–32^. A previous study revealed that vitamin E could reduce cancer cell growth by suppressing telomerase activity in ovarian cancer cells^33^. This result also accorded with another study showing that the intake of vitamin E was inversely associated with ovarian cancer (OR = 0.6; 95%CI, 0.5–0.8)^34^. Future more preclinical studies are therefore recommended to validate the efficacy of vitamin E for ovarian cancer prevention.

Strengths of this analysis include the use of a comprehensive collection of micro-nutrients GWASs, the large number of cases of ovarian cancer, the appraisal of the role of these micro-nutrients in various ovarian cancer subtypes, the unlikely weak instrument bias (i.e., F-statistics for respective genetic instruments across the 13 micronutrients ranged from 18 to 344), the employment of Mendelian randomization approach to minimize confounding and avoid reverse causation bias, and the usage of complementary analyses to provide additional evidence regarding the causal relationship.

There are several limitations to our analyses. First, the statistical power was not high enough in the analysis of some kinds of micro-nutrients; specifically, the SNPs of folate only explained a small portion of the variance (1%) in serum folate levels. Hence, we cannot rule out the possibility of overlooking weak associations between genetically predicted serum folate concentrations and ovarian cancer subtypes, and the same is true for other nutrients. In this case, larger GWASs of micro-nutrients are required for the construction of better genetic instruments. Second, we performed several statistical tests to explore the potential violation of MR assumptions. Despite the lack of indication of directional pleiotropy in the results with significance, we cannot robustly exclude the associations from being mediated through other causal pathways due to some underpowered statistical tests when a small number of genetic instruments were used (for example, MR-Egger). Third, due to the genetic studies largely limited in European ancestry, it might restrict the finding’s generalisability in other ancestral groups. Fourth, we should draw conclusions based on effect estimates and their CIs, rather than only based on statistical tests using a P value cut-off^35,36^. Given some point estimates with wider CIs, the effects of some micro-nutrients were not so precisely estimated, for example, the effect of magnesium on invasive epithelial ovarian (95% CI, 0.03–0.70) and the effect of vitamin E on low malignant potential tumours (95% CI, 0.06–0.76). Also, the point estimate of effect of β-carotene on invasive epithelial ovarian with the CI including the null (95% CI, 1.00–1.09) should be treated with more caution as well. Fifth, all models employed assumed linear relationships between micro-nutrients and ovarian cancer. Future studies focusing on nonlinear relationships of the concentration of micro-nutrients and the risk of epithelial ovarian cancer are warranted to elucidate potential underlying pathways. In spite of its limitations, our study certainly offers some insights into the correlation between micro-nutrients and ovarian cancer.

## Conclusion

In conclusion, we conducted the first comprehensive two-sample MR study to investigate whether the concentrations of thirteen micro-nutrients were related to the risk of epithelial ovarian cancer. An increased risk of invasive epithelial ovarian cancer was observed with a genetically higher concentration of β-carotene, whereas a decreased risk of invasive epithelial ovarian cancer was found with a higher concentration of magnesium. As for low malignant potential tumours, increased concentration of vitamin B12 could increase the risk of low malignant potential tumours, while increased concentrations of β-carotene and vitamin E could lower the risk of low malignant potential tumours.

## Methods

### Study design

**Figure 2:**
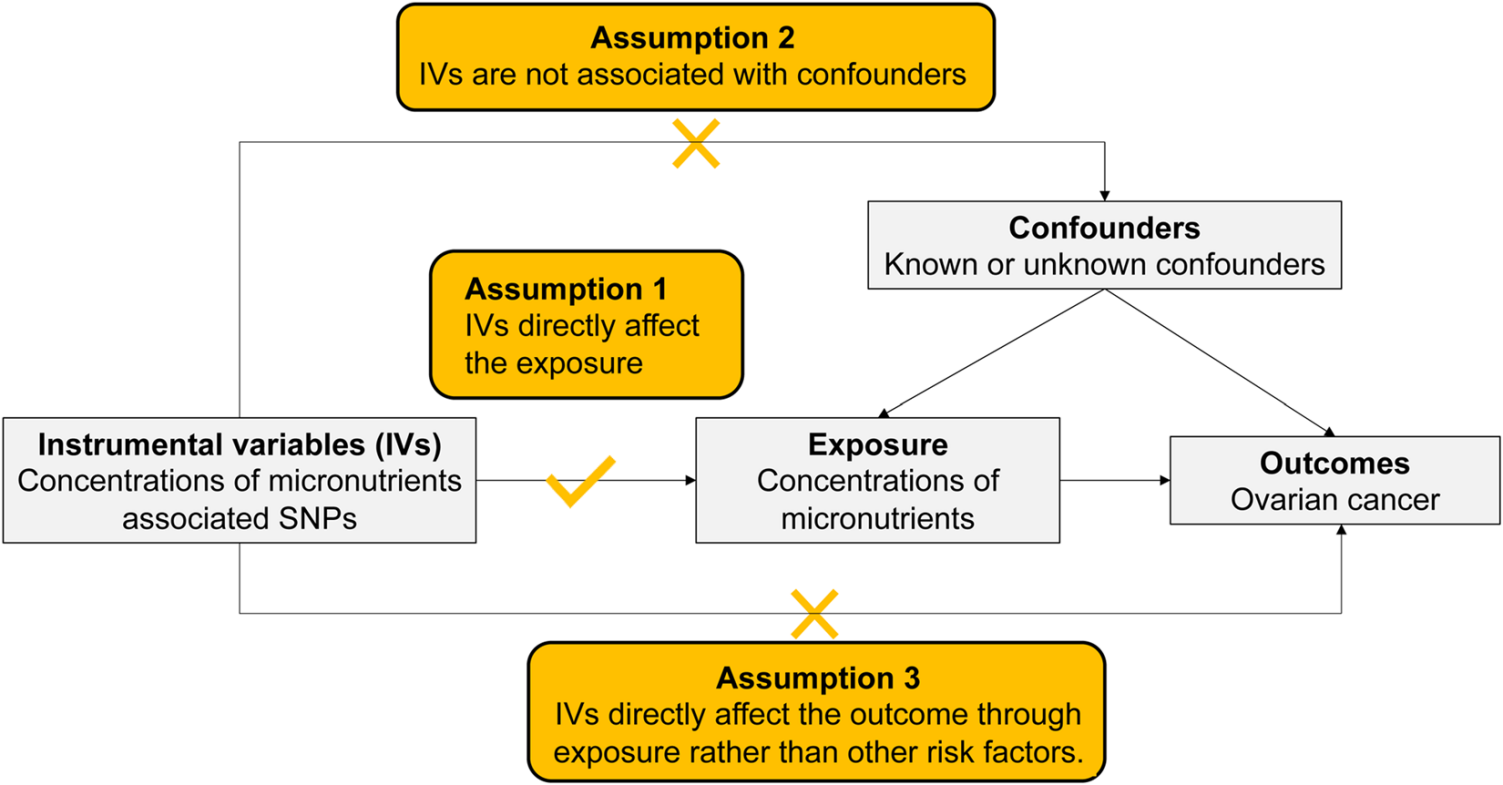
Schematic representation of an Mendelian randomization analysis of the association of micro-nutrients with ovarian cancer.

A Mendelian randomization study that examined whether single nucleotide polymorphisms (SNPs) associated with concentrations of micro-nutrients are causally linked to the risk of ovarian cancer. The unbiased causal relationship between exposures and disease outcomes can be estimated by using genetic instruments for exposures if the following assumptions are fulfilled: (1) the genetic instruments (one or more independent SNPs) are robustly related to the exposures concerned; (2) the genetic instruments are not associated with any possible confounding variables; (3) the genetic instruments influence outcomes only through the exposures (Fig. 2).

### Ovarian cancer population

Genotype data were obtained from the Illumina Custom Infinium array (OncoArray) project, a part of the Ovarian Cancer Association Consortium (OCAC) genome-wide association study (GWAS). There were 25,509 epithelial ovarian cancer (EOC) cases and 40,941 controls of European ancestry included in the summary genetic association data^37^. The OCAC OncoArray data comprised 63 genotyping project/case-control sets. From seven genotyping projects, 66,450 samples passed quality control. Analyses were performed for 40,941 controls, 22,406 invasive epithelial ovarian cancer cases, and 3,103 low malignant potential tumours cases. The invasive cases (n = 22,406) involved the following histotypes: high grade serous carcinoma (n = 13,037), low grade serous carcinoma (n = 1,012), mucinous carcinoma (n = 1,417), endometrioid carcinoma (n = 2,810), clear cell carcinoma (n = 1,366), and others (n = 2,764). The low malignant potential tumours (n = 3,103) included serous borderline tumours (n = 1,954) and mucinous borderline tumours (n = 1,149).

### Micro-nutrients GWAS sources

Published GWAS on micro-nutrients, including minerals and vitamins, were searched by using Genome-Wide Association Studies (GWAS) catalog (https://www.ebi.ac.uk/gwas) and Pubmed (https://www.ncbi.nlm.nih.gov/pubmed). Vitamin D was excluded because the role of vitamin D in ovarian cancer has been investigated by MR studies^38,39^. Vitamins B1, B2, B3, B5, B7, C, sulfur, iodine, chloride and fluoride were excluded because no genome-wide association studies have been conducted. Vitamin K, potassium, sodium, cobalt, chromium and molybdenum were also excluded for no genome-wide significant results^40–42^. In total, thirteen micro-nutrients with suitable genetic instruments were included in the analysis: calcium, magnesium, iron, copper, zinc, phosphorus, selenium, β-carotene, vitamin A, B6, B12, E and folate^26,40,43–51^.

### Genetic instrument selection

After obtaining effect estimates from relevant GWASs, SNPs were pruned for linkage disequilibrium at r^2^ < 0.1 from the lead SNP at a genome-wide significance level (P < 5 × 10^−8^). Then, the corresponding effect estimates and standard errors of the remaining independent SNPs were obtained from the ovarian cancer dataset. When an SNP was not available in the ovarian cancer dataset, the presence of a “proxy” SNP in linkage disequilibrium with this SNP at r^2^ > 0.8 was assessed using the Phase 3 (Version 5) of the 1000 Genomes Project sample data (identified using online tool SNiPa, available at http://snipa.helmholtz-muenchen.de/snipa3/). If such “proxy” SNP was not available in the ovarian cancer dataset, this SNP was excluded from the analysis.

### Statistical analyses

Estimates of the proportion of variance in each micro-nutrient explained by the genetic instruments (R^2^) and the strength of the association between the genetic instruments and micro-nutrients (F-statistics) were generated using methods previously described^52^. Inverse-variance-weighted (IVW) fixed effects models were used to generate effect estimates for micro-nutrients with 2 or 3 SNPs as instruments. IVW multiplicative random effects models were used if the number of SNPs was greater than 3^53^. Complementary analyses using the weighted median, MR-Egger regression, MR-robust adjusted profile score (MR-RAPS, an MR method for correcting for horizontal pleiotropy using robust adjusted profile scores^54^) and MR-Pleiotropy Residual Sum and Outlier (MR-PRESSO, an MR method for correcting pleiotropy residual sum and outlier)^55^. Directional pleiotropy statistics were conducted using MR-Egger, and the intercept term can provide a formal statistical test for pleiotropy. Additionally, whether the results were driven by individual influential SNPs was examined by using leave-one-out permutation analyses. MR analyses were conducted using the TwoSampleMR R packages^56^. A two-sided p-value of <0.05 was set as the threshold for significance. To avoid inference based simply on P-value thresholds, the direction and strength of effect for each association, together with the corresponding P-value, were presented^35,36^. All analyses were conducted in R version 3.6.1.

### Ethics approval and informed consent

All procedures performed in studies involving human participants were in accordance with the ethical standards of the institutional committee and with the 1964 Helsinki declaration. The protocol of OCAC was approved by each of the Ethics Committees of the participating institutions. The specific study reported here was approved by the Zhejiang University Ethics Committee. Written informed consent was obtained from all individual participants included in the study. All the methods were carried out in accordance with the approved guidelines.

## Supplementary information

Supplementary Information.

## Data Availability

The datasets analyzed in this study are publicly available summary statistics.
