# Supplementary material for: Appraising the role of circulating concentrations of micro-nutrients in epithelial ovarian cancer risk: A Mendelian randomization analysis
Source: Sci Rep. 2020 Apr 30;10:7356. doi: 10.1038/s41598-020-63909-5 (PMC7193611; doi:10.1038/s41598-020-63909-5)
Supplement: Supplementary file 1 — Supplementary Information. [file 41598_2020_63909_MOESM1_ESM.pdf]

SUPPLEMENTARY INFORMATION

Appraising the role of circulating concentrations of micro-nutrients in epithelial ovarian cancer risk: A Mendelian randomization analysis

Yan Guo[1,2], Yunlong Lu[1], Hongchuan Jin[2*]

[1]School of Medicine, Zhejiang University, Hangzhou 310058, China; [2]Laboratory of Cancer Biology, Key Lab of Biotherapy in Zhejiang, Sir Run Run Shaw Hospital, Medical School of Zhejiang University, Hangzhou 310000, China

Keywords

Mendelian randomization, nutrition, ovarian cancer, causal inference

S1 Data. Summary genetic association data used to perform analyses

Phenotype	SNP	Gene	Eaf	Chr	Position	Effect_allele	Other_allele	Beta	Se	P-value
Fe	rs1800562	HFE	0.0427	6	26092913	A	G	0.328	0.016	2.72E-97
Fe	rs1799945	HFE	0.828	6	26090951	C	G	-0.189	0.010	1.10E-81
Fe	rs855791	TMPRSS6	0.3877	22	37462936	A	G	-0.181	0.007	1.32E-139
Cu	rs1175550	SMIM1	0.7813	1	3691528	A	G	-0.198	0.032	5.03E-10
Cu	rs2769264	SELENBP1	0.161	1	151344741	G	T	0.313	0.034	2.63E-20
Zn	rs1532423	CA1	0.3708	8	86268313	A	G	0.178	0.026	6.40E-12
Zn	rs2120019	PPCDC	0.2078	15	75334184	C	T	-0.287	0.033	1.55E-18
Zn	rs4826508	NBDY	0.5596	X	56811695	T	C	0.21	0.030	1.40E-12
Ca	rs1801725	CASR	0.1451	3	122003757	T	G	0.071	0.004	8.90E-86
Ca	rs1570669	CYP24A1	0.6819	20	52774427	A	G	-0.018	0.003	9.10E-12
Ca	rs1550532	DGKD	0.3171	2	234264848	C	G	0.018	0.003	8.20E-11
Ca	rs7481584	CARS	0.2922	11	3029089	A	G	-0.018	0.003	1.20E-10
Ca	rs780094	GCKR	0.4105	2	27741237	T	C	0.017	0.003	1.30E-10
Ca	rs7336933	DGKH	0.1491	13	42559076	A	G	-0.022	0.004	9.10E-10
Ca	rs10491003	LINC00709	0.1014	10	9328651	T	C	0.027	0.005	4.80E-09
Mg	rs4072037	MUC1	0.4523	1	155162067	C	T	-0.01	0.001	2.01E-36
Mg	rs7965584	RP11-654D12.2	0.2694	12	90305779	G	A	-0.007	0.001	1.05E-16
Mg	rs3925584	DCDC1	0.5099	11	30760335	C	T	-0.006	0.001	5.20E-16
Mg	rs11144134	TRPM6	0.9235	9	77499796	T	C	-0.011	0.001	8.21E-15
Mg	rs13146355	SHROOM3	0.5676	4	77412140	G	A	-0.005	0.001	6.27E-13
Mg	rs448378	MECOM	0.4821	3	169100899	G	A	-0.004	0.001	1.25E-08
P	rs1697421	ALPL	0.509	1	21823292	T	C	0.044	0.005	3.47E-16
P	rs9469578	IHPK3	0.9304	6	33706479	C	T	-0.064	0.010	5.15E-10
P	rs947583	RP11-394G3.2	0.2336	6	136133659	C	T	0.035	0.006	2.19E-09

P	rs2970818	C12orf4	0.0746	12	4606168	A	T	0.052	0.010	4.04E-08
Se	rs921943	DMGDH	0.2952	5	78316476	T	C	0.246	0.023	9.40E-28
Se	rs7700970	BHMT	0.3171	5	78411324	T	C	0.212	0.024	1.72E-18
Vitamin A	rs10882272	FFAR4	0.3807	10	95348182	C	T	-0.03	0.004	6.51E10-15
Vitamin A	rs1667255	TTR	0.3757	18	29187279	C	A	0.03	0.004	6.35E-14
β-carotene	rs6420424	PKD1L2	0.4632	16	81242102	A	G	0.155	0.022	6.50E-13
β-carotene	rs8044334	PKD1L2	0.341	16	81248935	G	T	0.109	0.015	9.30E-13
β-carotene	rs11645428	PKD1L2	0.3628	16	81258896	A	G	-0.129	0.015	1.50E-17
β-carotene	rs6564851	BCO1	0.495	16	81264597	G	T	0.149	0.015	1.60E-24
Vitamin B6	rs4654748	ALPL	0.5	1	21786068	C	T	-1.45	0.280	8.30E-18
Vitamin B12	rs602662	FUT2	0.596	19	49206985	A	G	0.16	0.006	2.40E-139
Vitamin B12	rs34324219	TCN1	0.881	11	59623378	C	A	0.21	0.009	1.10E-111
Vitamin B12	rs34528912	TCN1	0.0361	11	59631535	T	C	0.17	0.022	2.10E-15
Vitamin B12	rs117456053	TCN1	0.976	11	59616831	G	A	0.16	0.027	1.90E-09
Vitamin B12	rs1801222	CUBN	0.593	10	17156151	G	A	0.11	0.008	2.30E-42
Vitamin B12	rs56077122	TRDMT1	0.335	10	17207015	A	C	0.087	0.009	4.80E-21
Vitamin B12	rs2336573	G220R	0.031	19	8367709	T	C	0.32	0.020	8.40E-59
Vitamin B12	rs1131603	TCN2	0.055	22	31018975	C	T	0.17	0.018	1.10E-21
Vitamin B12	rs5753231	TCN2	0.064	22	31003069	C	T	0.064	0.011	7.50E-10
Vitamin B12	rs41281112	CLYBL	0.948	13	100518634	C	T	0.17	0.014	8.90E-35
Vitamin B12	rs1141321	MUT	0.627	6	49412433	C	T	0.061	0.006	3.60E-26
Vitamin B12	rs3742801	ABCD4	0.294	14	74759006	T	C	0.045	0.006	1.70E-13
Vitamin B12	rs2270655	MMAA	0.941	4	146576418	G	C	0.066	0.009	2.20E-13
Vitamin B12	rs12272669	MMACHC	0.0022	11	71392610	A	G	0.51	0.088	3.00E-09
Vitamin B12	rs7788053*	FUT6	0.254	7	86773722	A	G	0.046	0.007	1.70E-10
Vitamin E	rs964184	BUD13/ZNF259	0.15	11	116648917	G	C	0.04	0.010	7.80E-12

Vitamin E	rs2108622	CYP4F2	0.21	19	15990431	T	C	0.03	0.010	1.40E-10
Vitamin E	rs11057830	SCARB1	0.15	12	125307053	A	G	0.03	0.010	8.20E-09
Folate	rs1801133	MTHFR	0.668	1	11856378	G	A	0.1	0.009	3.40E-27
Folate	rs17421511	MTHFR	0.827	1	11857788	G	A	0.098	0.012	1.80E-15
Folate	rs652197	FOLR3	0.179	11	71849741	C	T	0.069	0.011	2.50E-10

*SNP not available in the ovarian cancer dataset

S1 Plots. Scatter plots for findings showing strong or suggestive evidence of association in IVW analyses

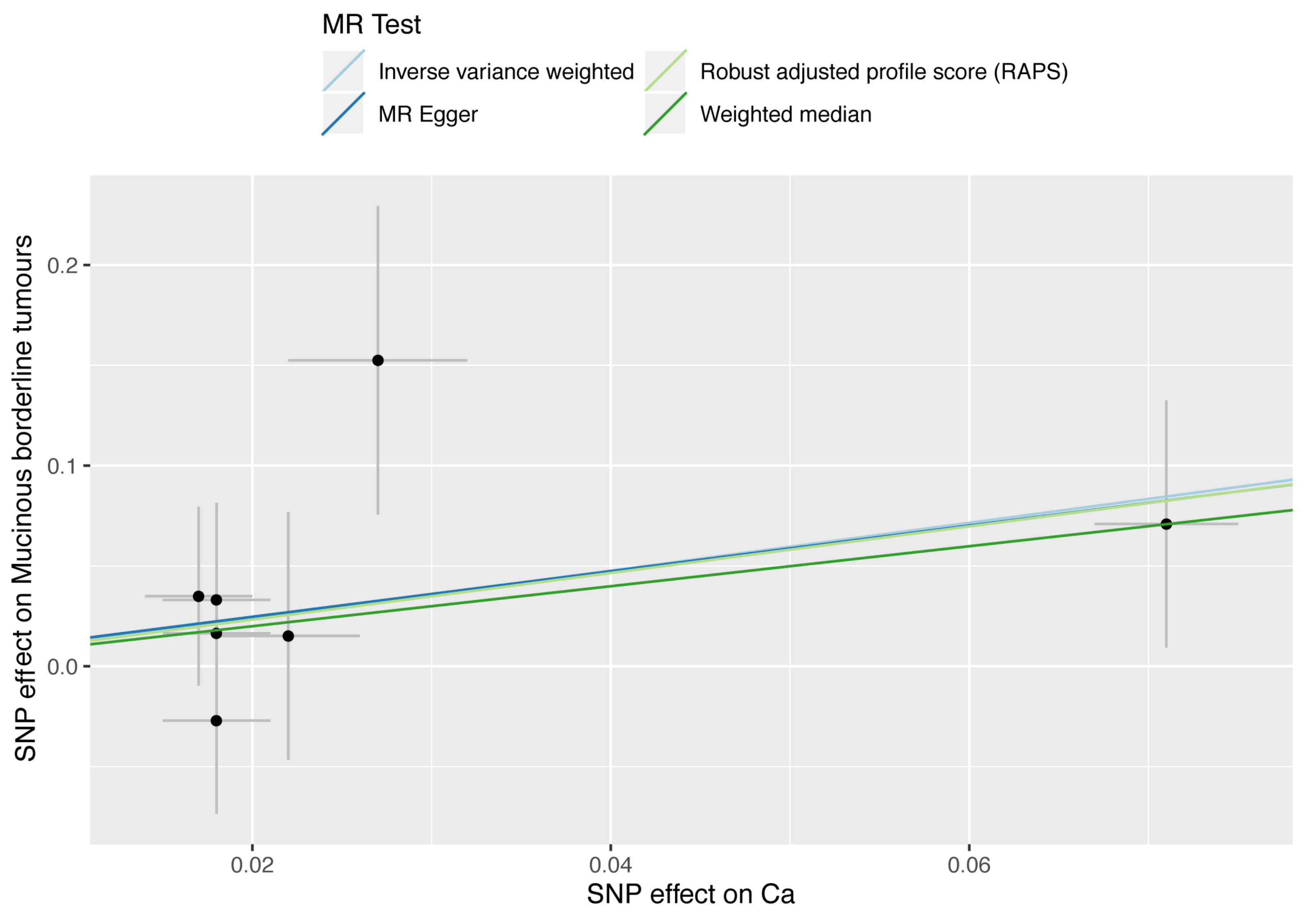
MR Test
Inverse variance weighted
Robust adjusted profile score (RAPS)
MR Egger
Weighted median
0.2
0.1
0.0
0.02
0.04
0.06
SNP effect on Mucinous borderline tumours
SNP effect on Ca

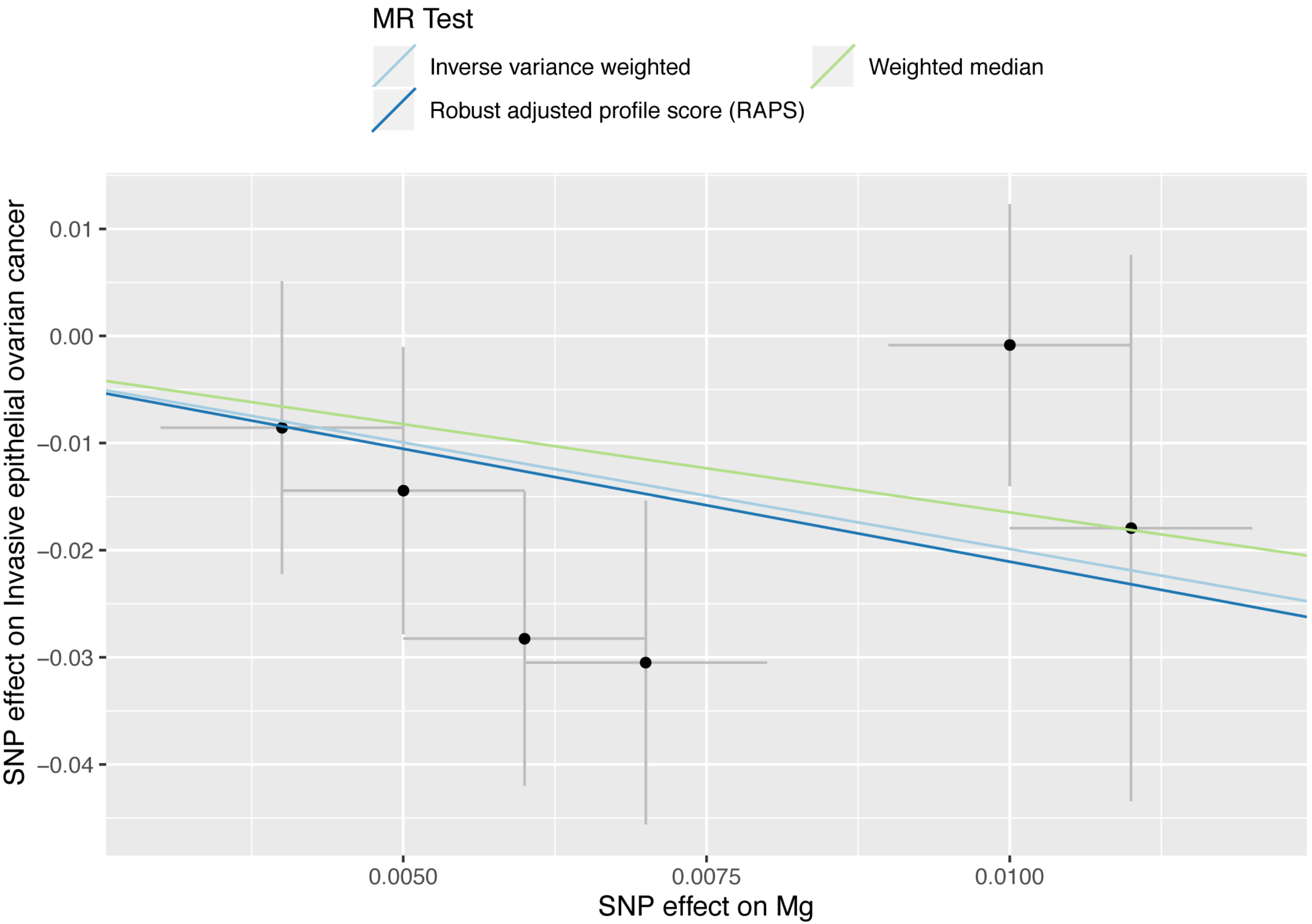
MR Test
Inverse variance weighted
Weighted median
Robust adjusted profile score (RAPS)
0.01
0.00
−0.01
−0.02
−0.03
−0.04
SNP effect on Invasive epithelial ovarian cancer
0.0050
0.0075
0.0100
SNP effect on Mg

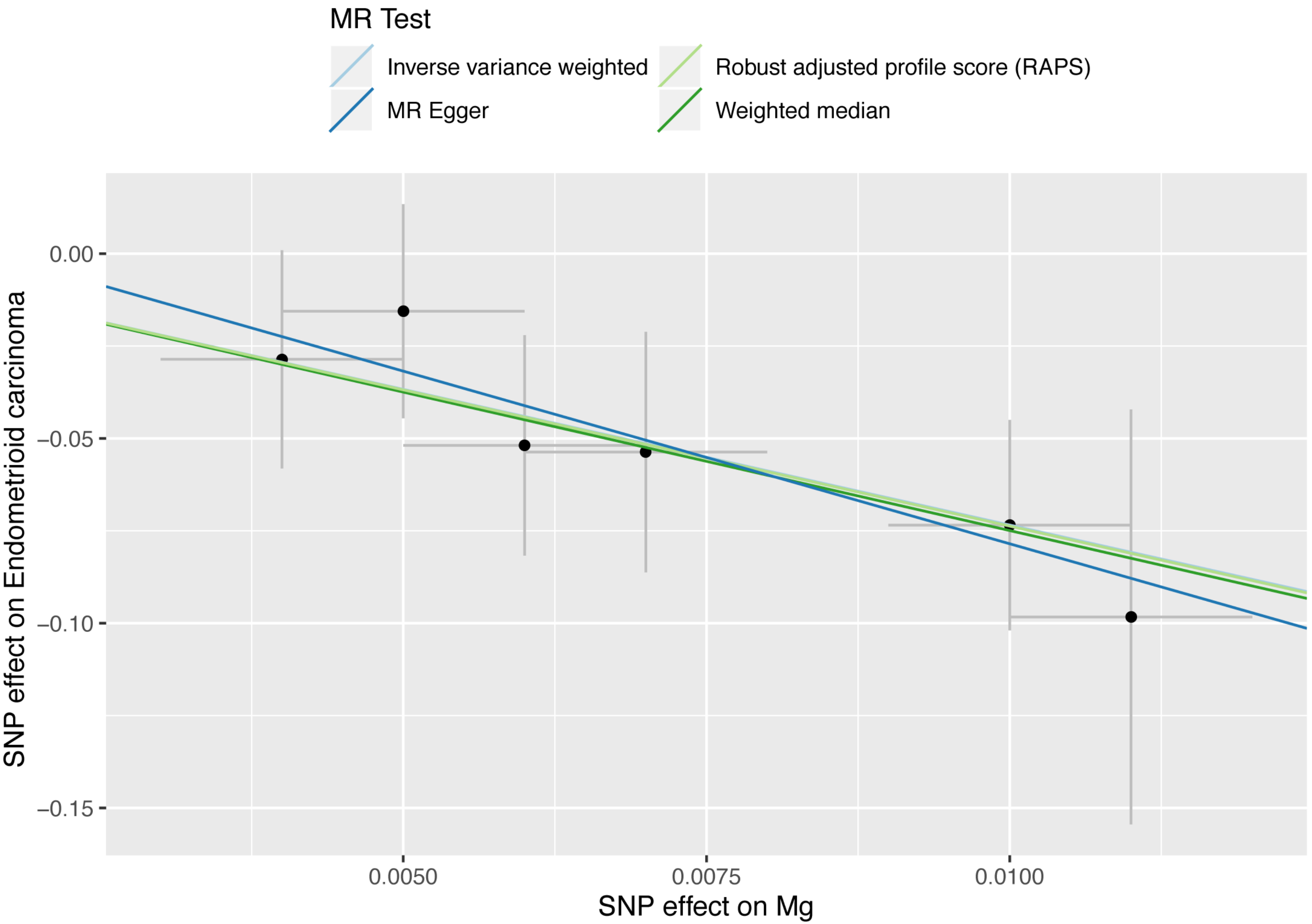

MR Test
Inverse variance weighted
Robust adjusted profile score (RAPS)
MR Egger
Weighted median
0.00
−0.05
−0.10
−0.15
SNP effect on Endometrioid carcinoma
0.0050
0.0075
0.0100
SNP effect on Mg

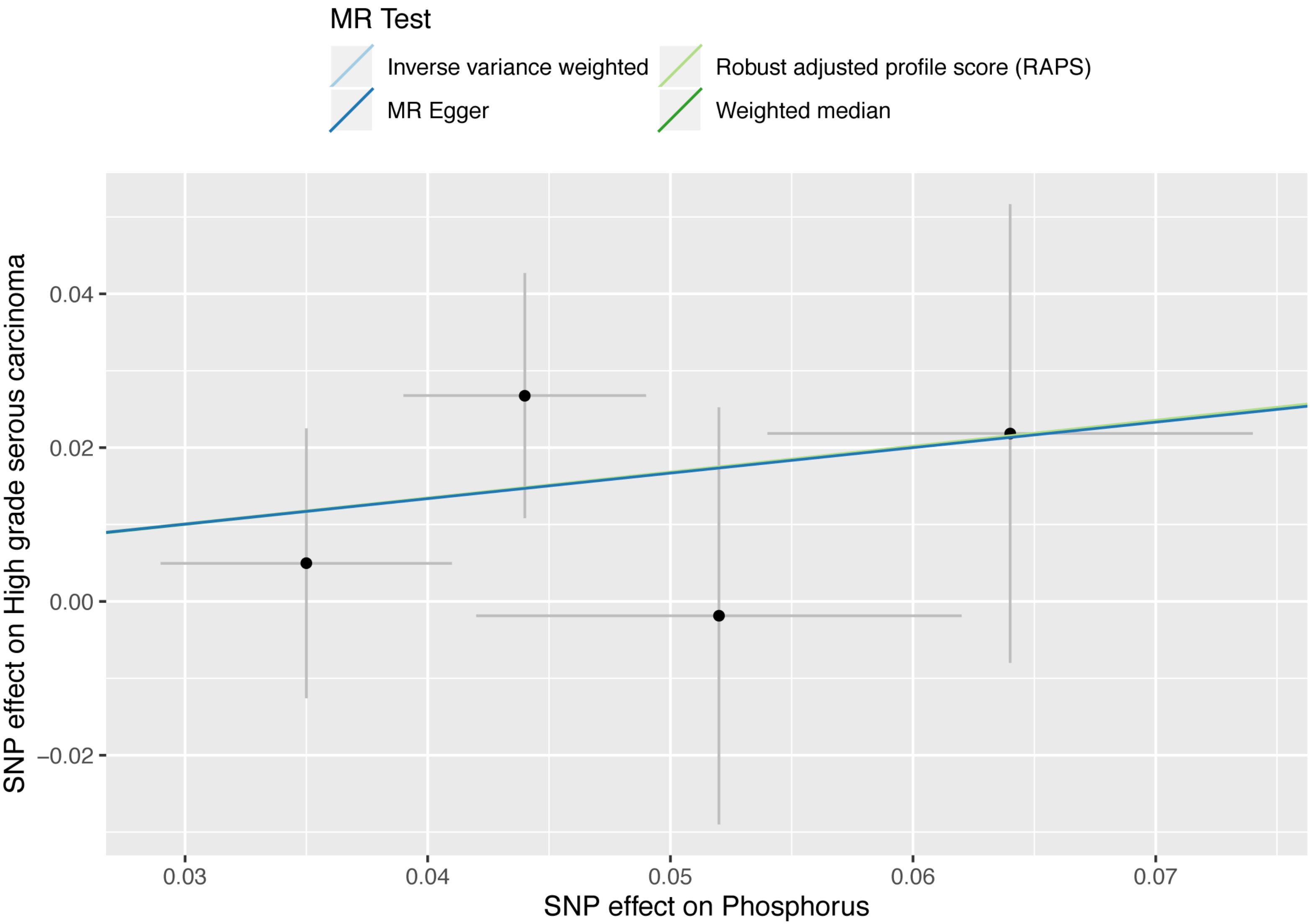

MR Test
Inverse variance weighted
MR Egger
Robust adjusted profile score (RAPS)
Weighted median
0.04
0.02
0.00
−0.02
0.03
0.04
0.05
0.06
0.07
SNP effect on High grade serous carcinoma
SNP effect on Phosphorus

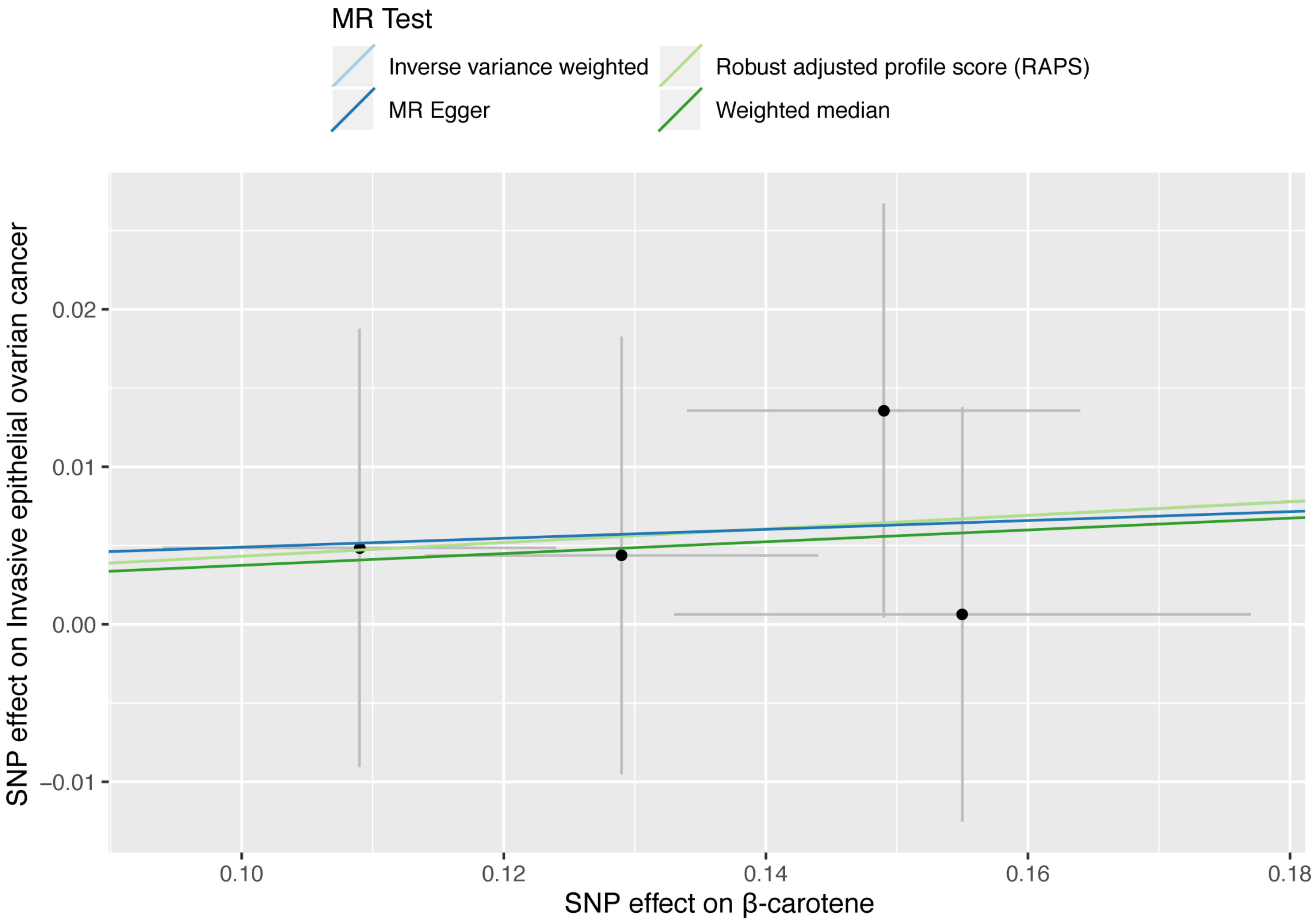
MR Test
Inverse variance weighted
Robust adjusted profile score (RAPS)
MR Egger
Weighted median
0.02
0.01
0.00
−0.01
0.10
0.12
0.14
0.16
0.18
SNP effect on Invasive epithelial ovarian cancer
SNP effect on β-carotene

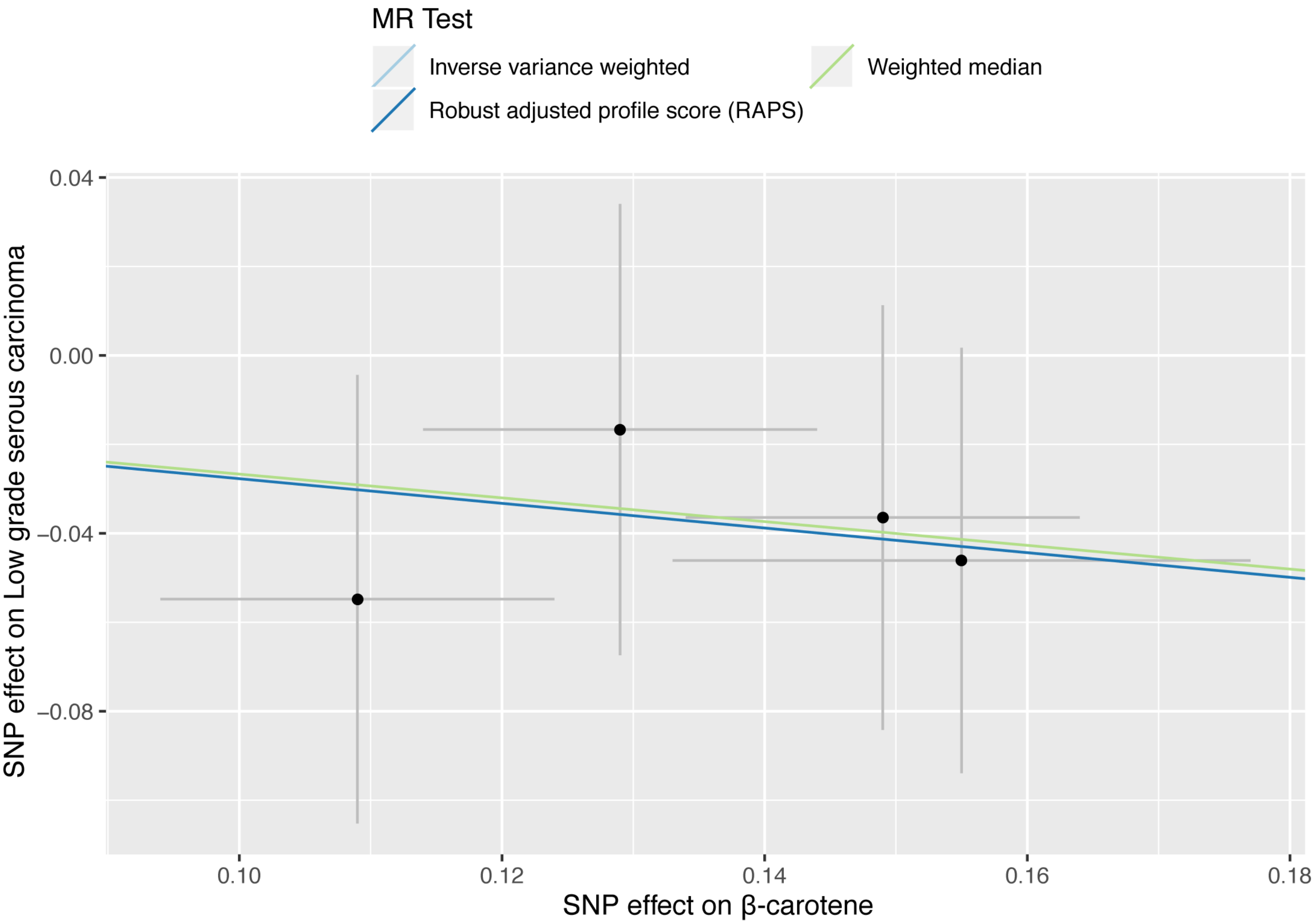

MR Test
Inverse variance weighted
Weighted median
Robust adjusted profile score (RAPS)
0.04
0.00
−0.04
−0.08
SNP effect on Low grade serous carcinoma
0.10
0.12
0.14
0.16
0.18
SNP effect on β-carotene

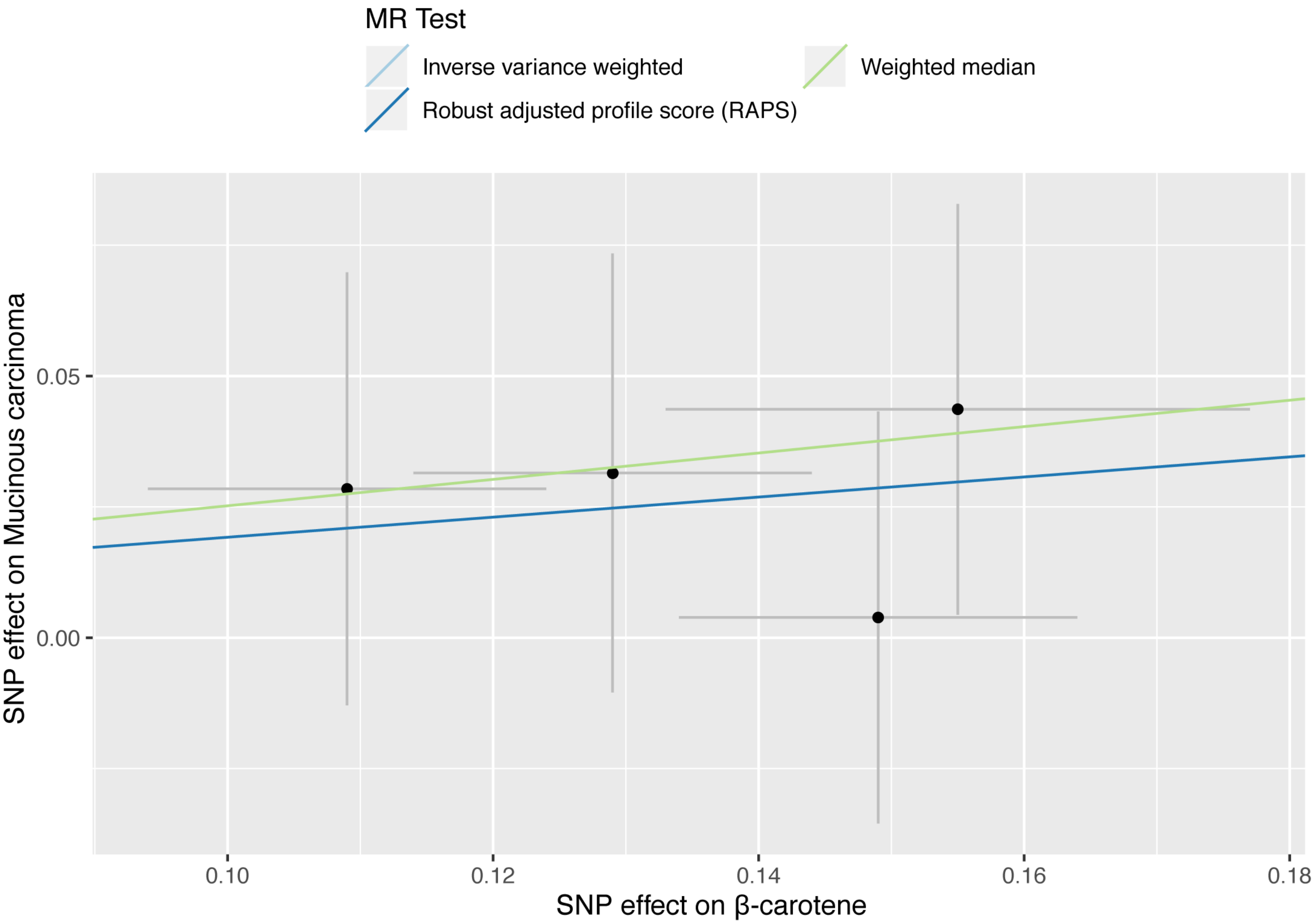
MR Test
Inverse variance weighted
Weighted median
Robust adjusted profile score (RAPS)
0.05
0.00
SNP effect on Mucinous carcinoma
0.10
0.12
0.14
0.16
0.18
SNP effect on β-carotene

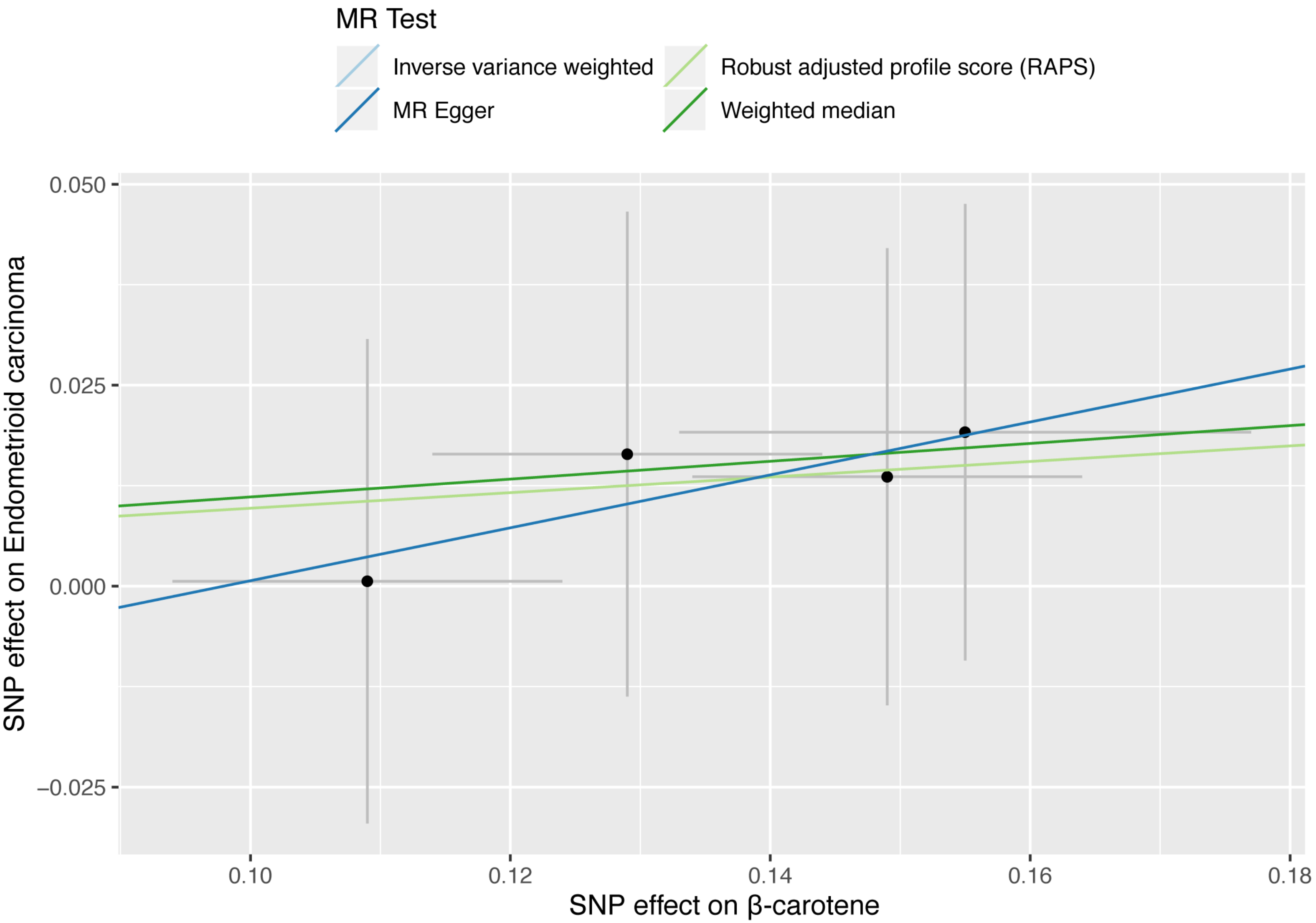

MR Test
Inverse variance weighted
Robust adjusted profile score (RAPS)
MR Egger
Weighted median
0.050
0.025
0.000
−0.025
SNP effect on Endometrioid carcinoma
0.10
0.12
0.14
0.16
0.18
SNP effect on β-carotene

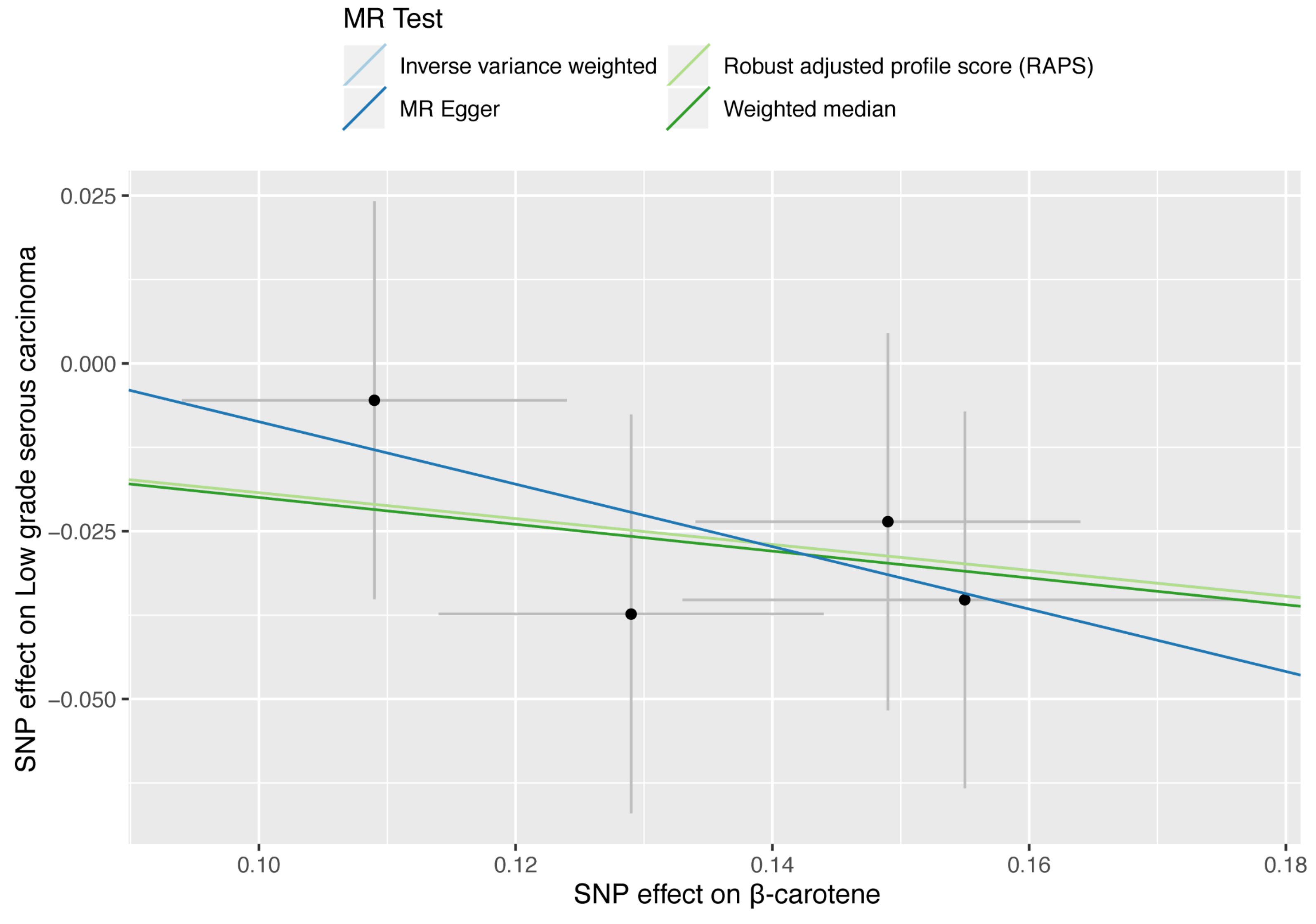
MR Test
Inverse variance weighted
MR Egger
Robust adjusted profile score (RAPS)
Weighted median
0.025
0.000
−0.025
−0.050
0.10
0.12
0.14
0.16
0.18
SNP effect on β-carotene
SNP effect on Low grade serous carcinoma

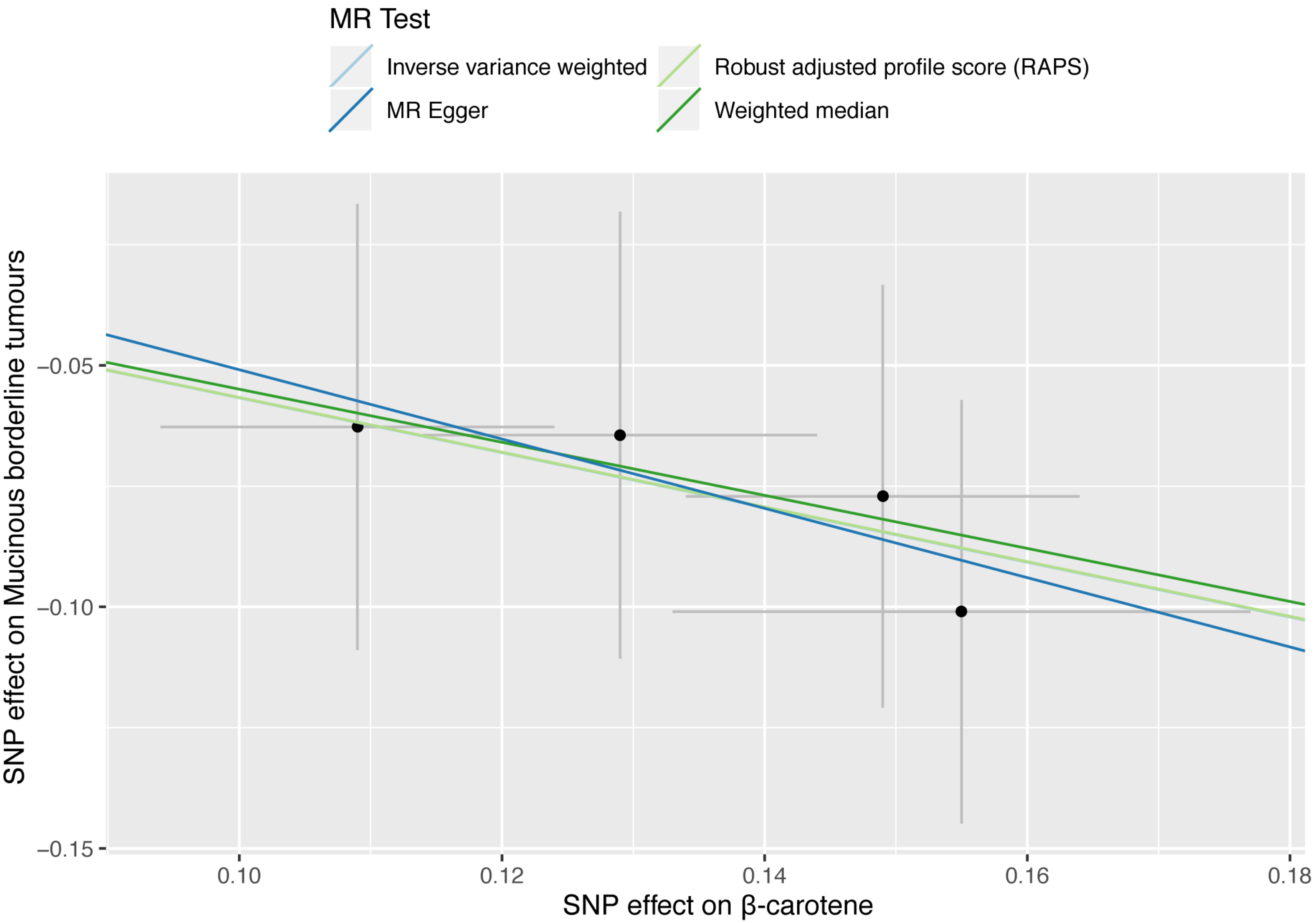
MR Test
Inverse variance weighted
MR Egger
Robust adjusted profile score (RAPS)
Weighted median
SNP effect on Mucinous borderline tumours
−0.05
−0.10
−0.15
0.10
0.12
0.14
0.16
0.18
SNP effect on β-carotene

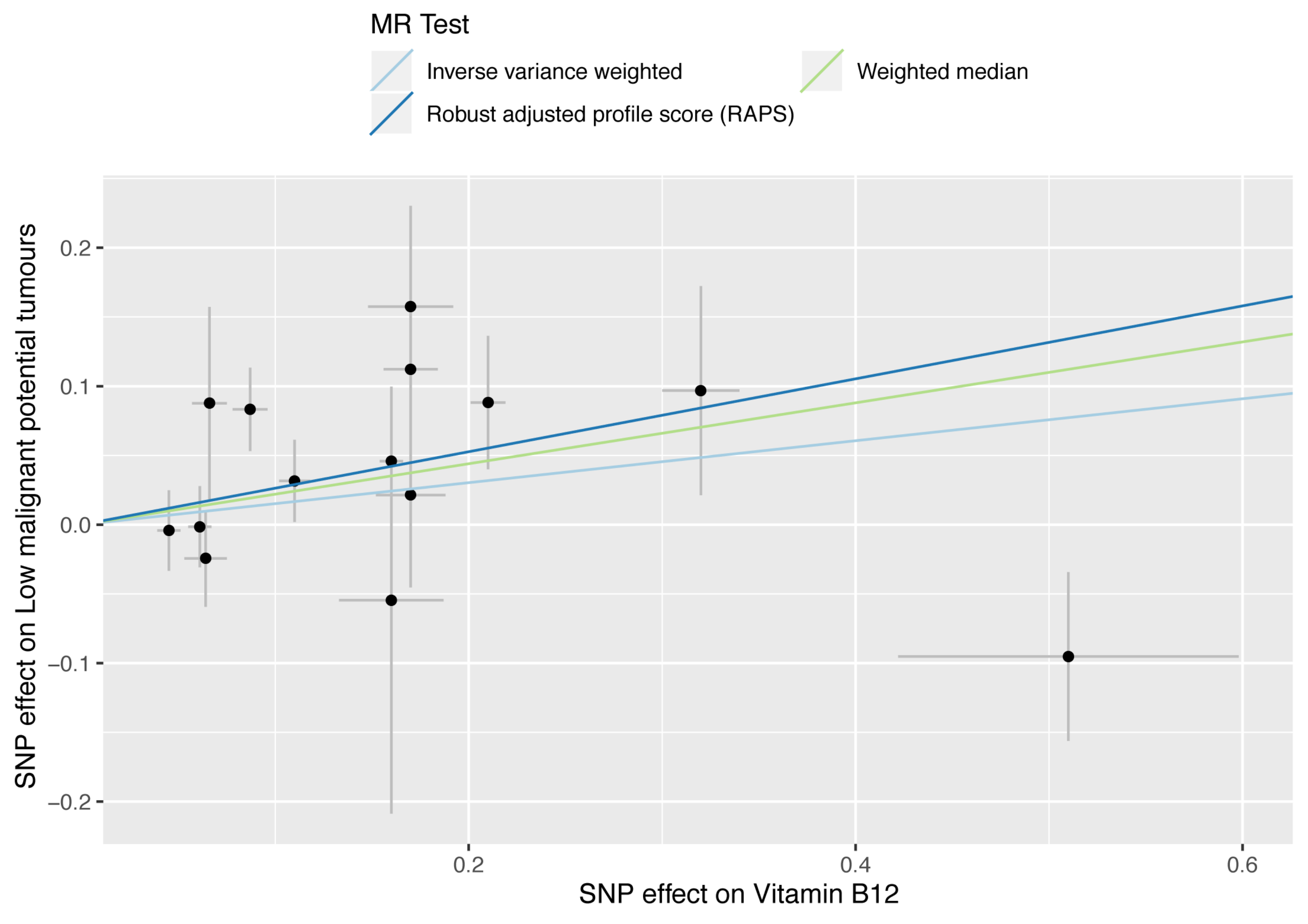
MR Test
Inverse variance weighted
Weighted median
Robust adjusted profile score (RAPS)
SNP effect on Low malignant potential tumours
0.2
0.1
0.0
−0.1
−0.2
0.2
0.4
0.6
SNP effect on Vitamin B12

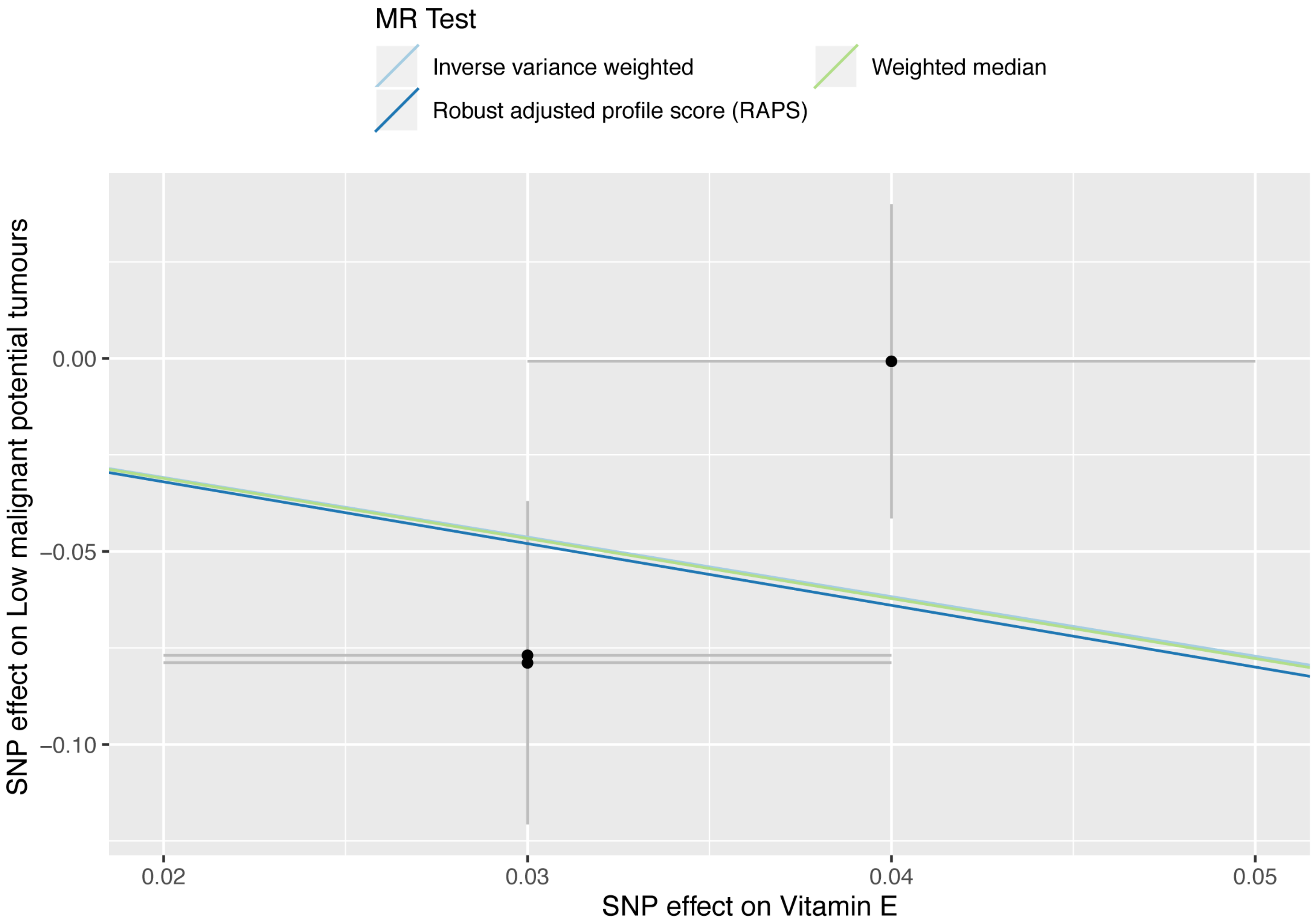

MR Test
Inverse variance weighted
Weighted median
Robust adjusted profile score (RAPS)
0.00
−0.05
−0.10
0.02
0.03
0.04
0.05
SNP effect on Vitamin E
SNP effect on Low malignant potential tumours

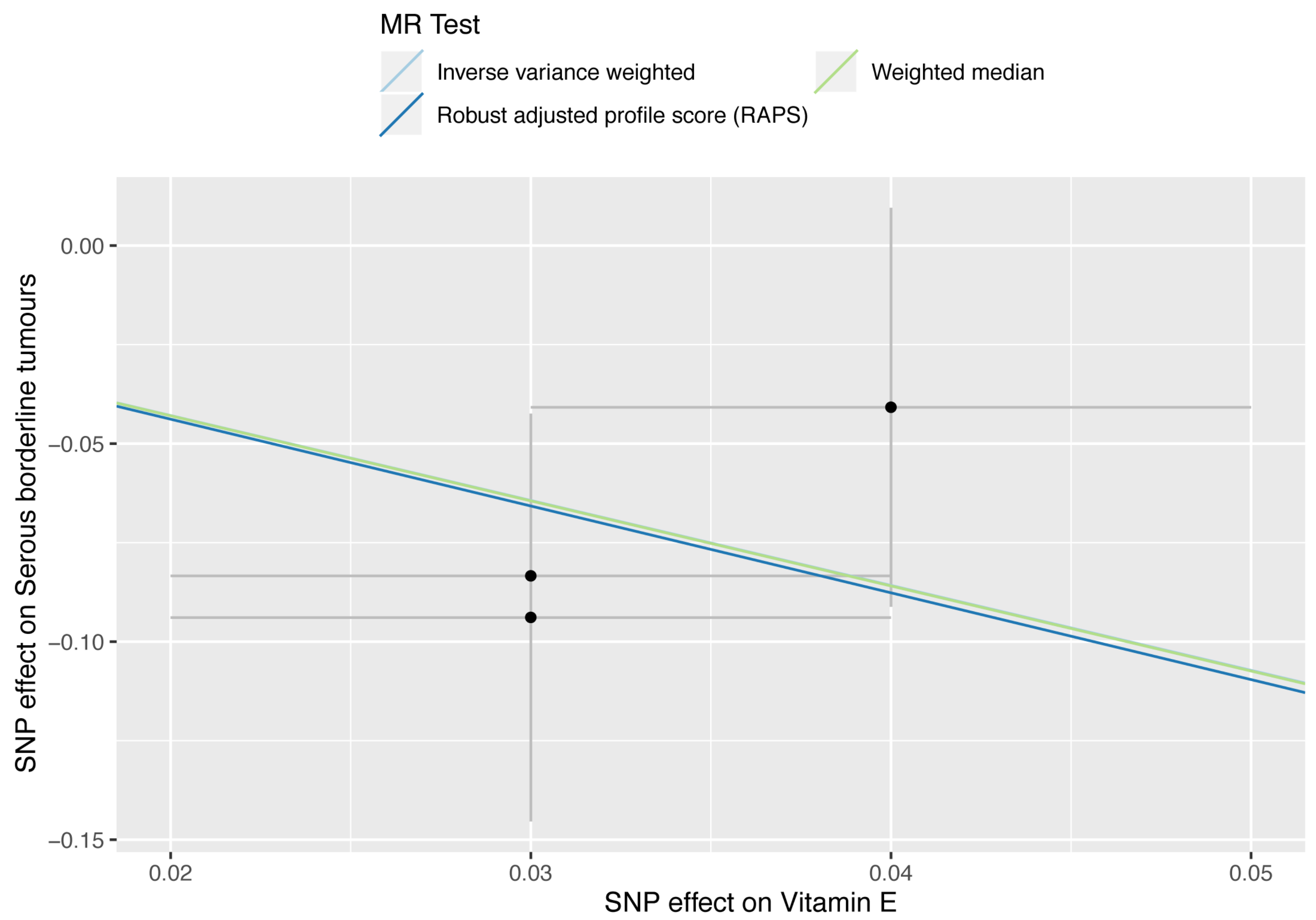

MR Test
Inverse variance weighted
Weighted median
Robust adjusted profile score (RAPS)
0.00
−0.05
−0.10
−0.15
SNP effect on Serous borderline tumours
0.02
0.03
0.04
0.05
SNP effect on Vitamin E

S2 Plots. Leave-one-out permutation analyses

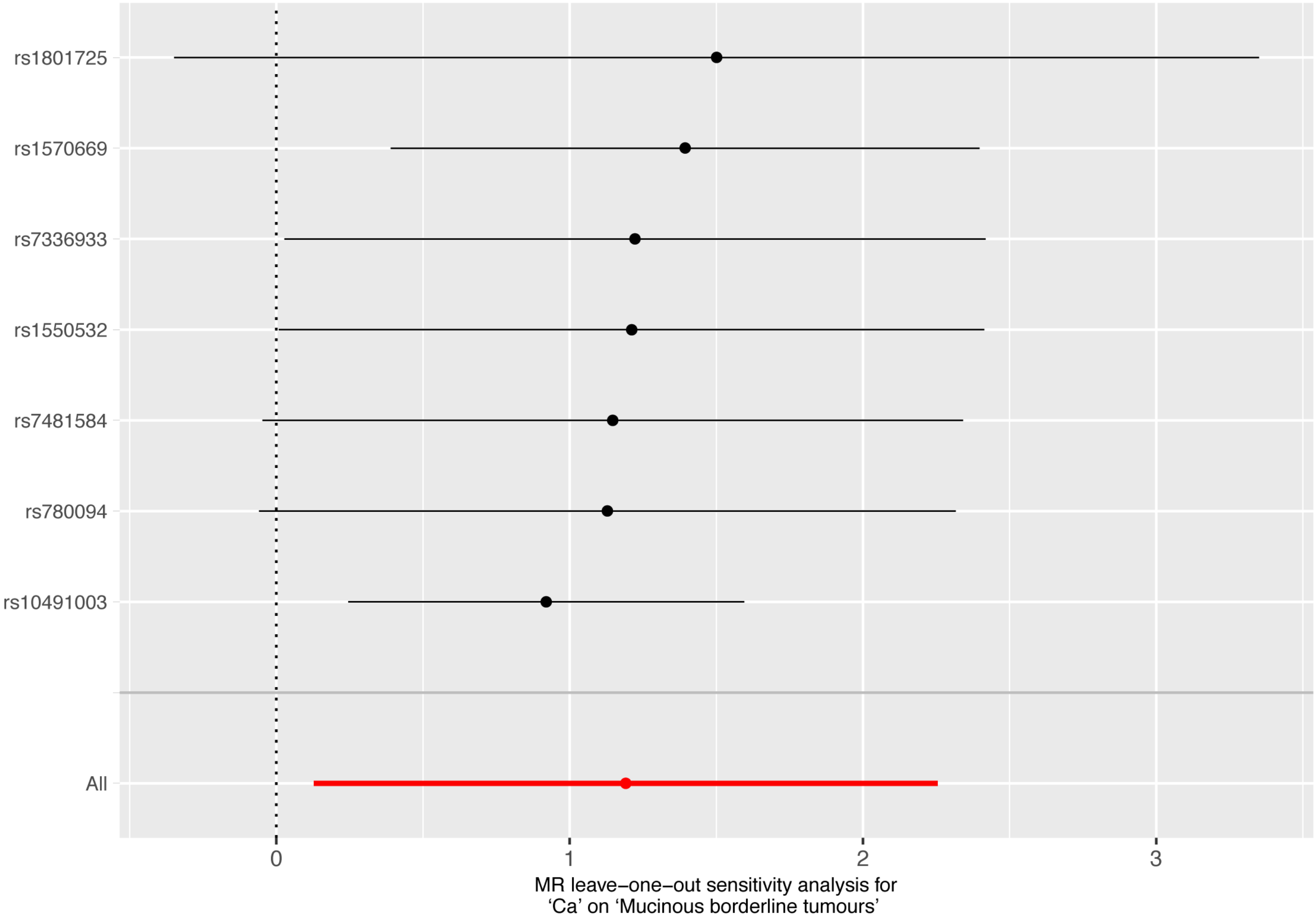

rs1801725
rs1570669
rs7336933
rs1550532
rs7481584
rs780094
rs10491003
All
0
1
2
3
MR leave−one−out sensitivity analysis for
'Ca' on 'Mucinous borderline tumours'

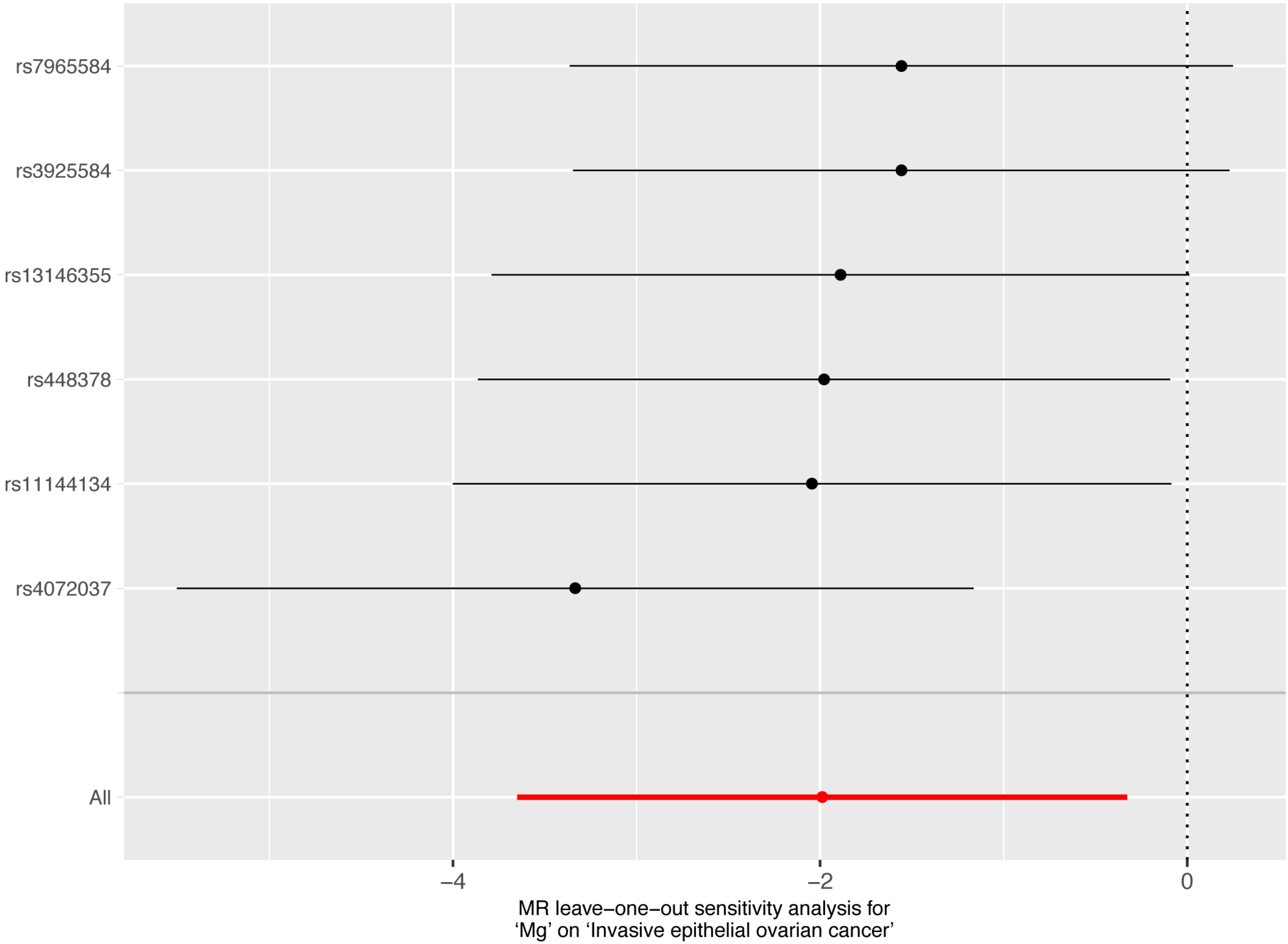

rs7965584
rs3925584
rs13146355
rs448378
rs11144134
rs4072037
All
−4
−2
0
MR leave−one−out sensitivity analysis for
'Mg' on 'Invasive epithelial ovarian cancer'

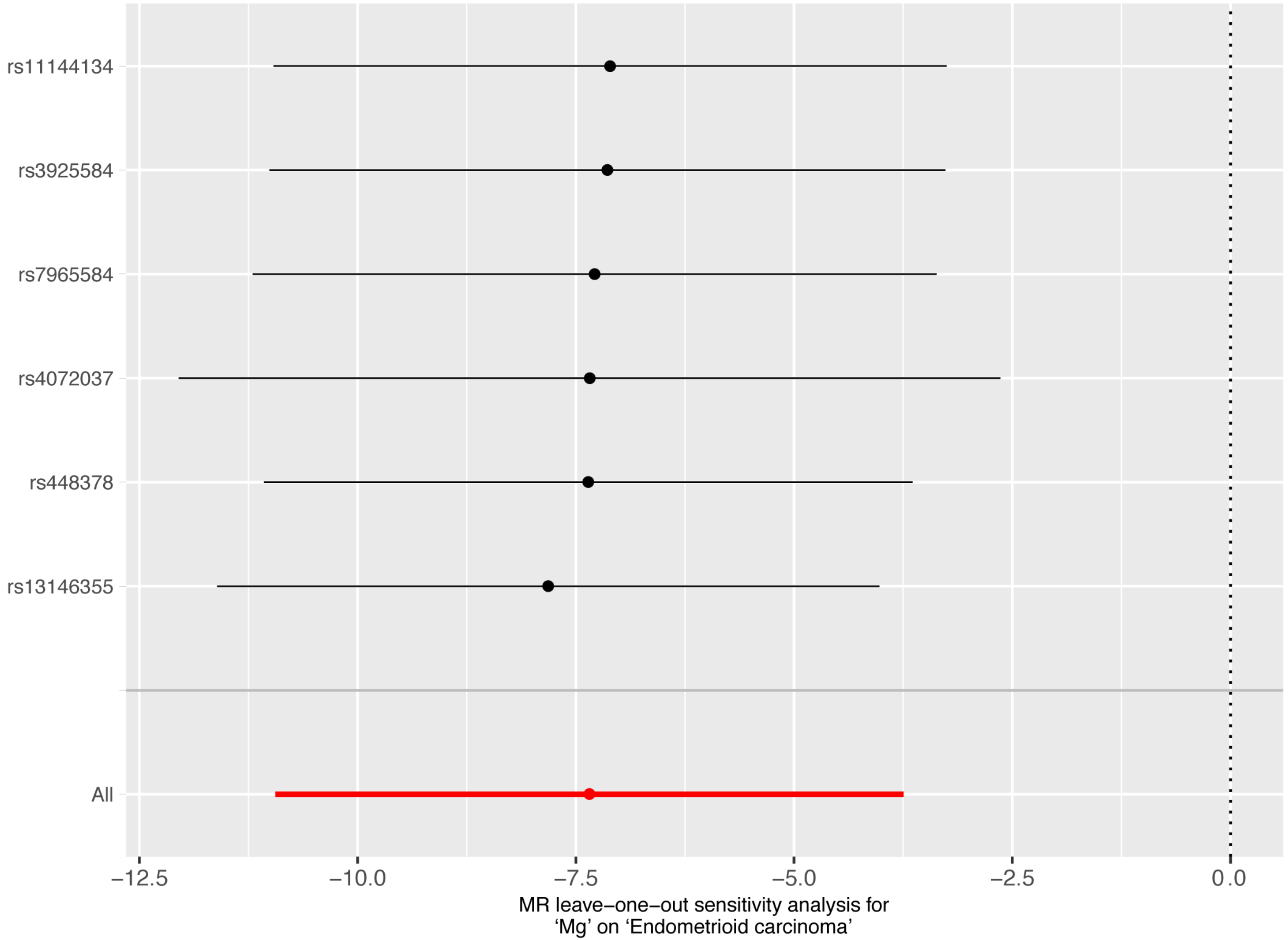
rs11144134
rs3925584
rs7965584
rs4072037
rs448378
rs13146355
All
−12.5
−10.0
−7.5
−5.0
−2.5
0.0
MR leave−one−out sensitivity analysis for
'Mg' on 'Endometrioid carcinoma'

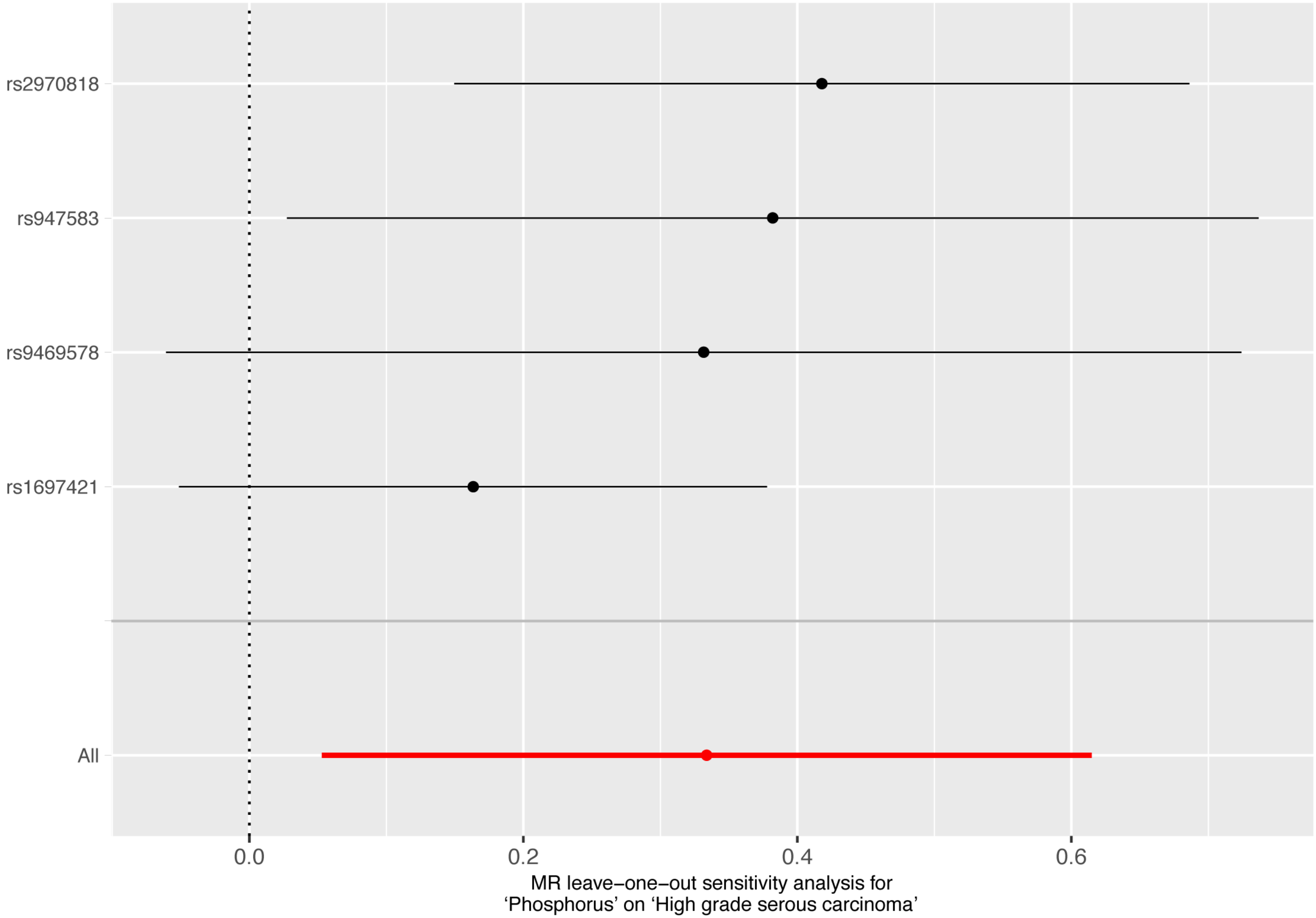
rs2970818
rs947583
rs9469578
rs1697421
All
0.0
0.2
0.4
0.6
MR leave−one−out sensitivity analysis for
'Phosphorus' on 'High grade serous carcinoma'

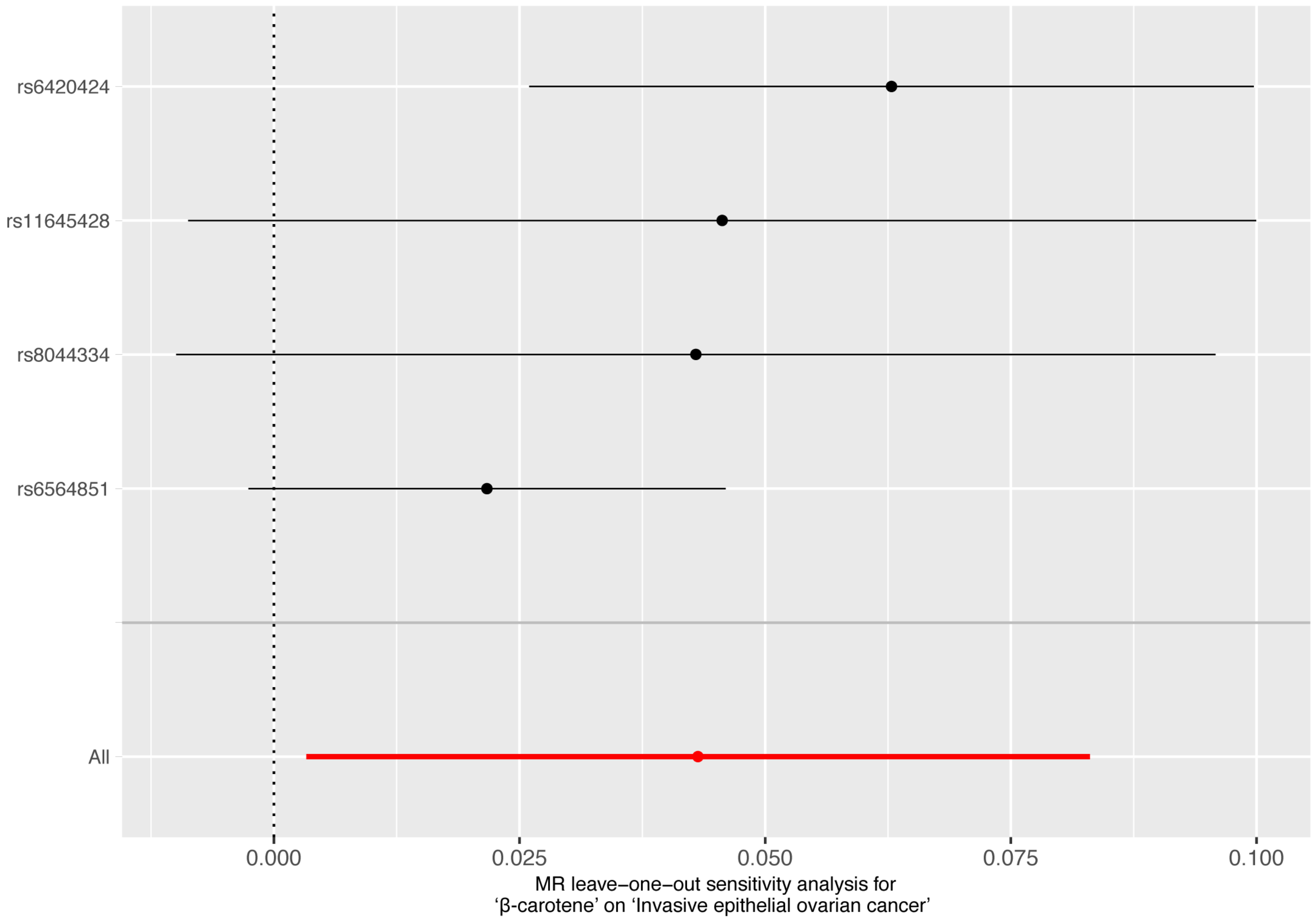
rs6420424
rs11645428
rs8044334
rs6564851
All
0.000
0.025
0.050
0.075
0.100
MR leave−one−out sensitivity analysis for
'β-carotene' on 'Invasive epithelial ovarian cancer'

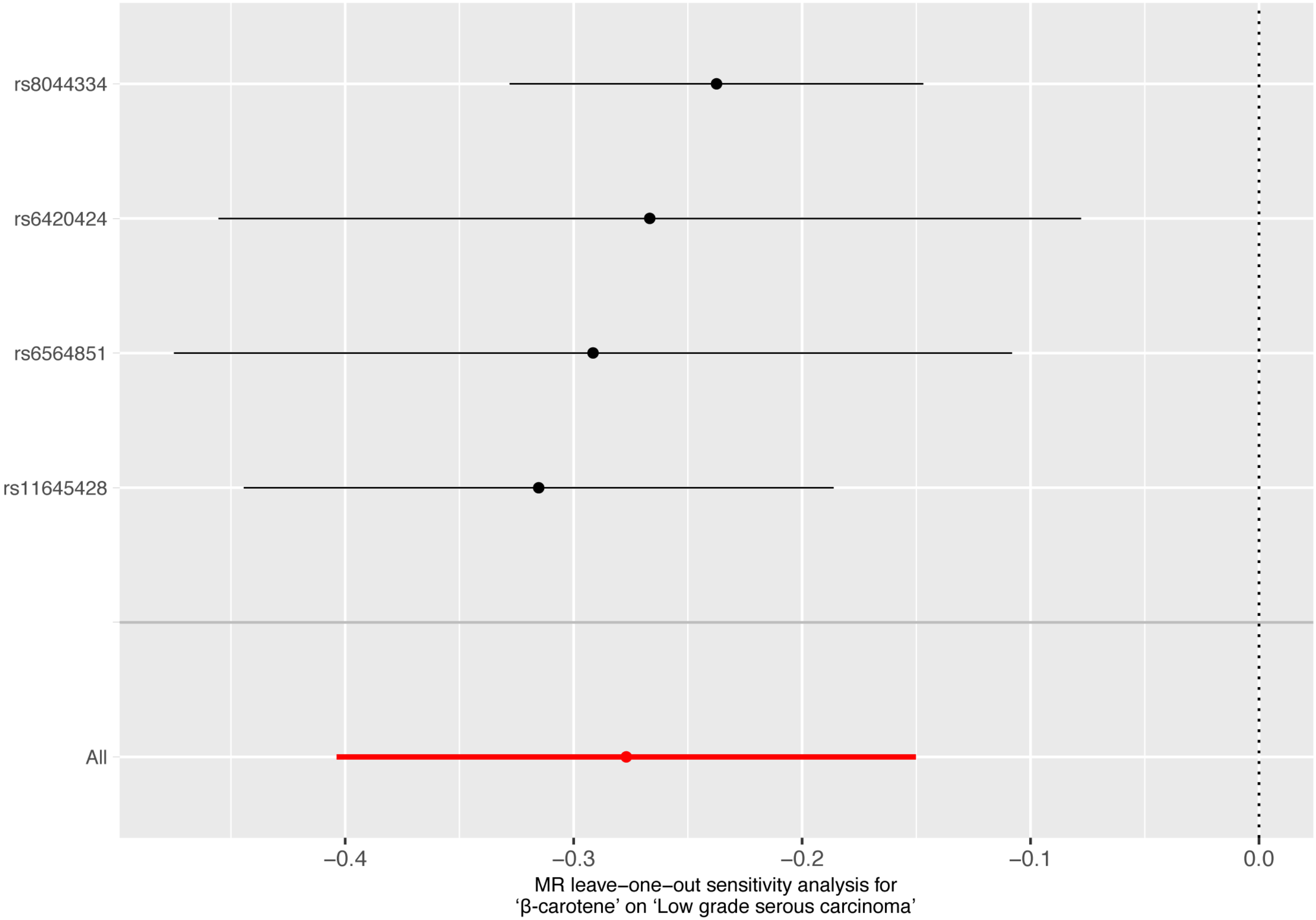
rs8044334
rs6420424
rs6564851
rs11645428
All
−0.4
−0.3
−0.2
−0.1
0.0
MR leave−one−out sensitivity analysis for
'β-carotene' on 'Low grade serous carcinoma'

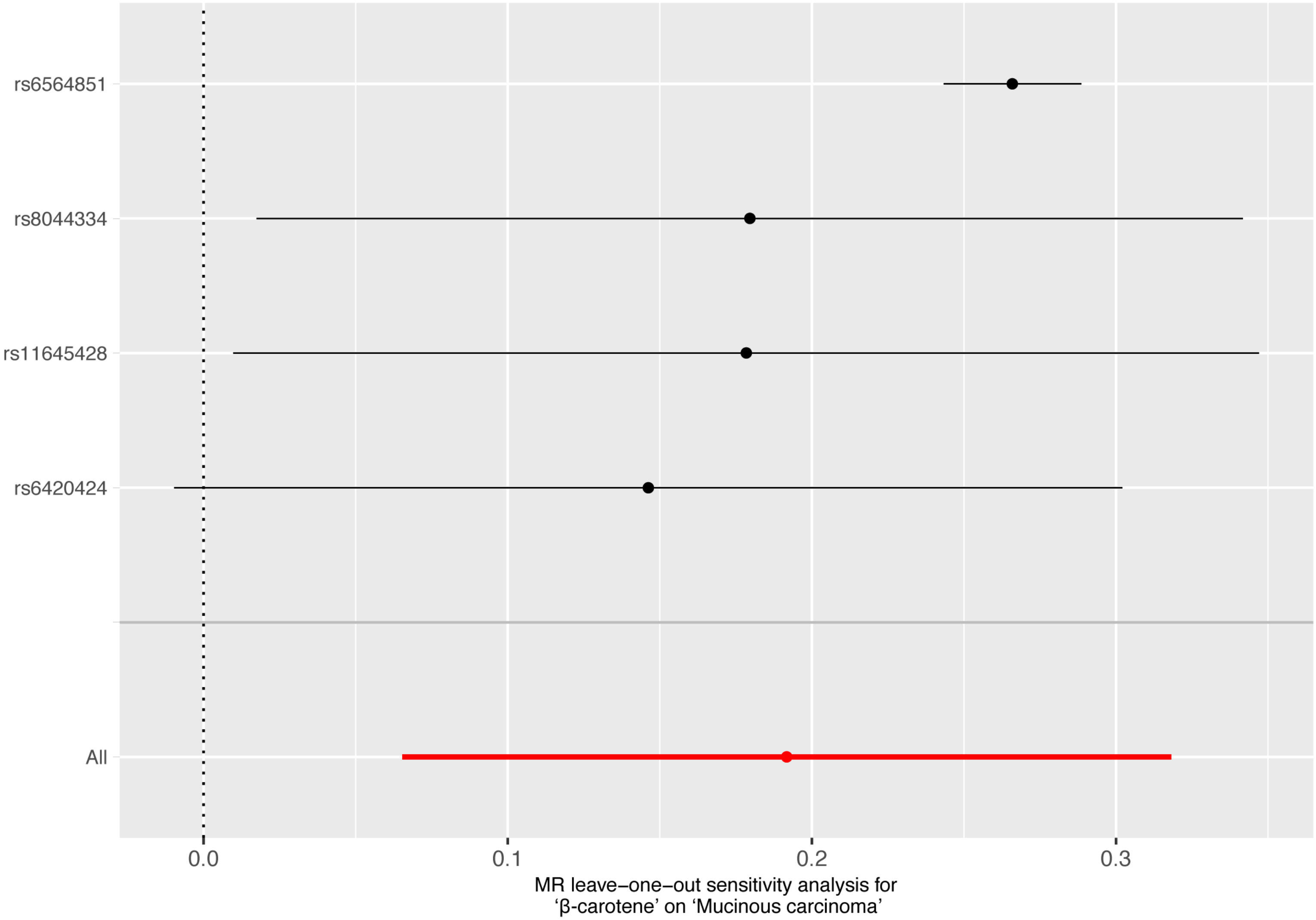
rs6564851
rs8044334
rs11645428
rs6420424
All
0.0
0.1
0.2
0.3
MR leave−one−out sensitivity analysis for
'β-carotene' on 'Mucinous carcinoma'

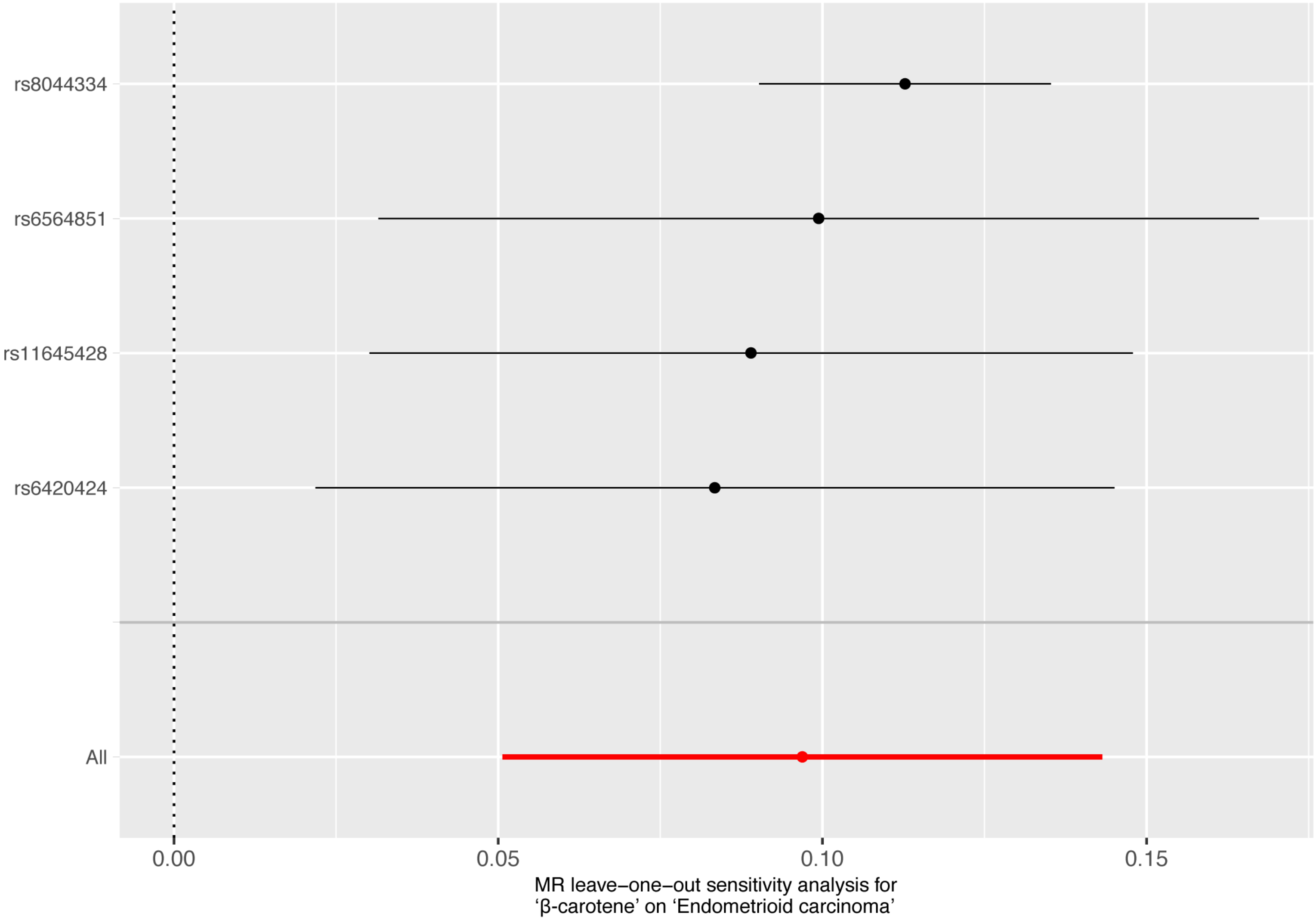
rs8044334
rs6564851
rs11645428
rs6420424
All
0.00
0.05
0.10
0.15
MR leave−one−out sensitivity analysis for
'β-carotene' on 'Endometrioid carcinoma'

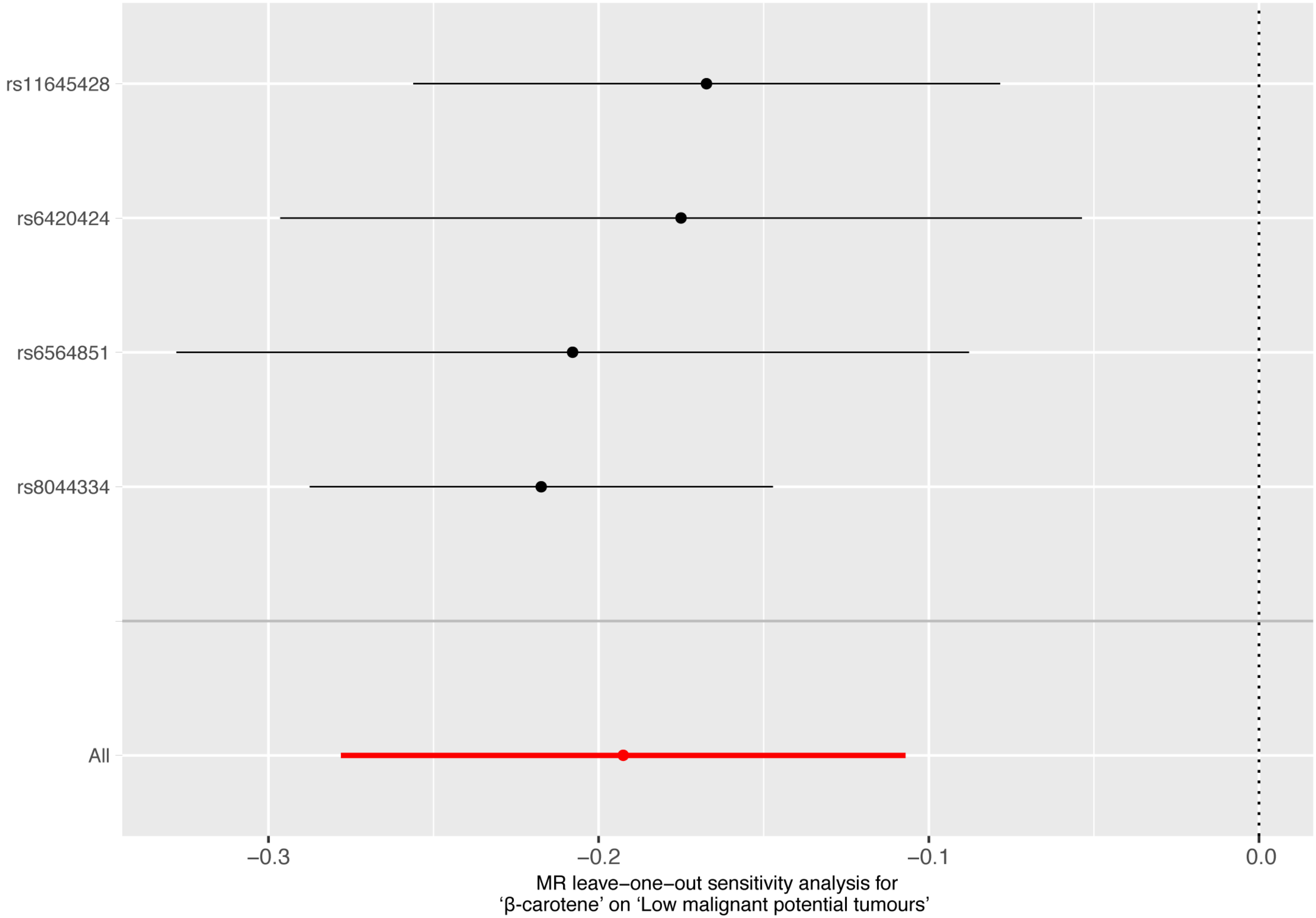
rs11645428
rs6420424
rs6564851
rs8044334
All
−0.3
−0.2
−0.1
0.0
MR leave−one−out sensitivity analysis for
'β-carotene' on 'Low malignant potential tumours'

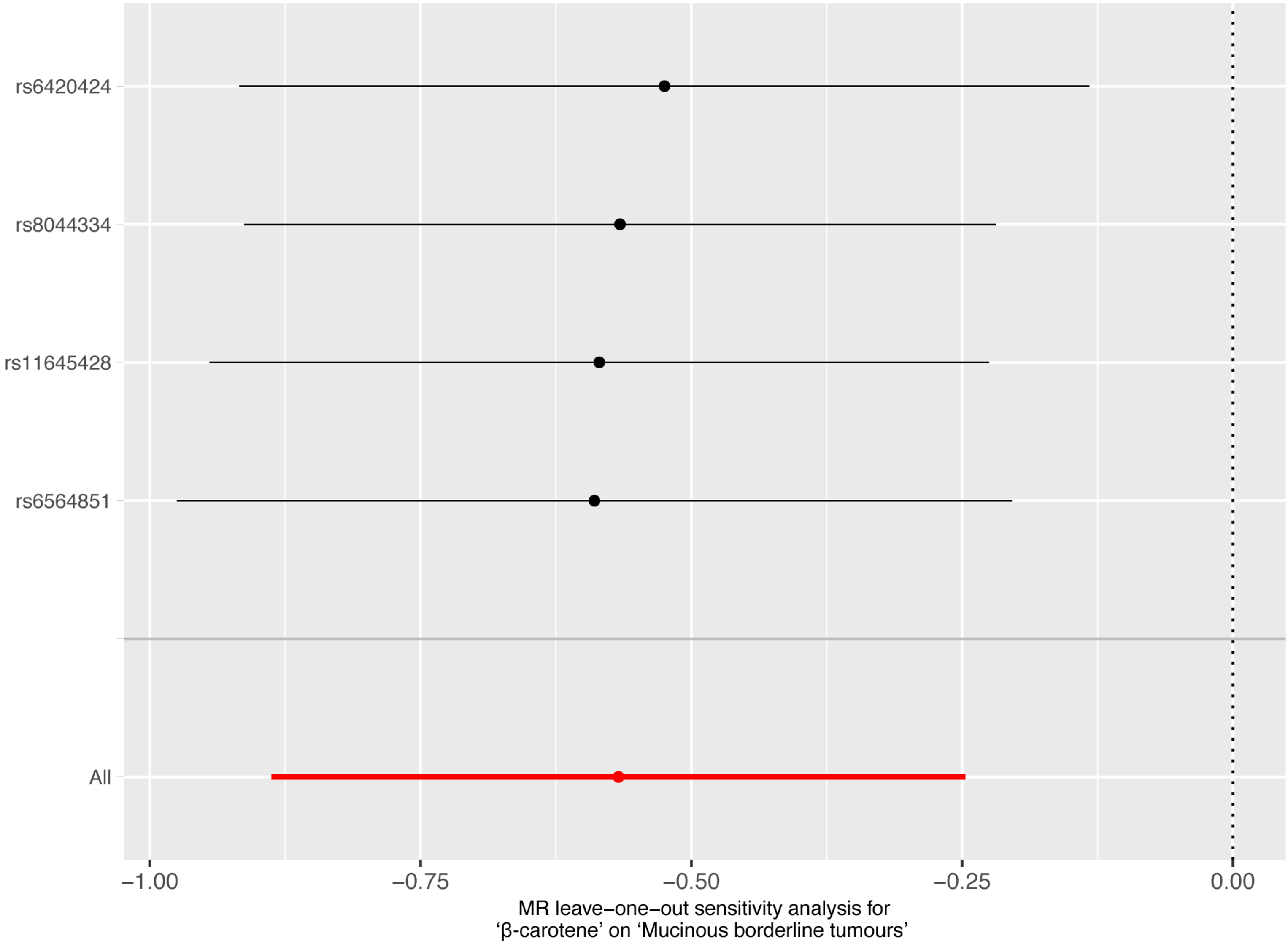

rs6420424
rs8044334
rs11645428
rs6564851
All
−1.00
−0.75
−0.50
−0.25
0.00
MR leave−one−out sensitivity analysis for
'β-carotene' on 'Mucinous borderline tumours'

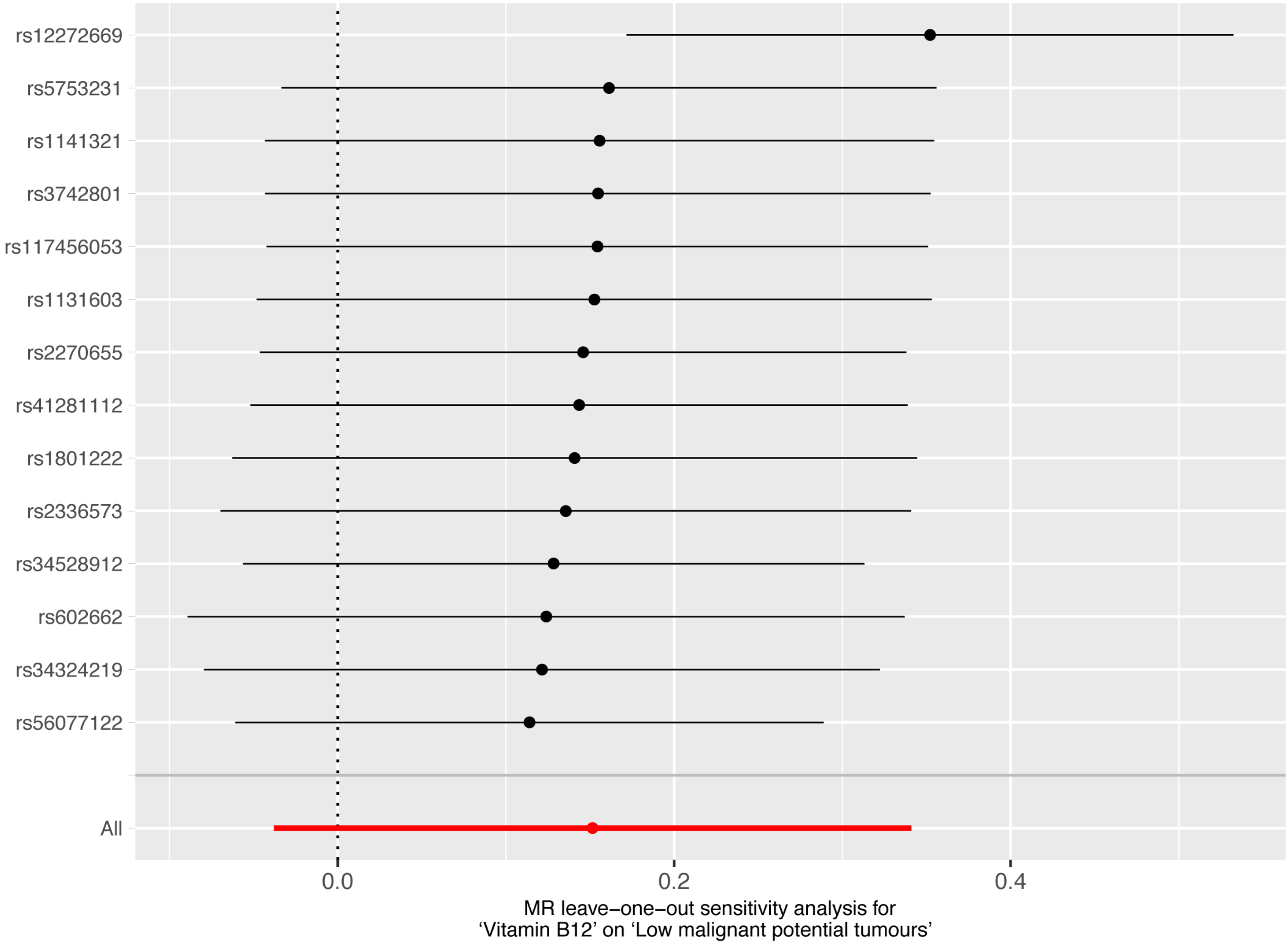
rs12272669
rs5753231
rs1141321
rs3742801
rs117456053
rs1131603
rs2270655
rs41281112
rs1801222
rs2336573
rs34528912
rs602662
rs34324219
rs56077122
All
0.0
0.2
0.4
MR leave−one−out sensitivity analysis for
'Vitamin B12' on 'Low malignant potential tumours'

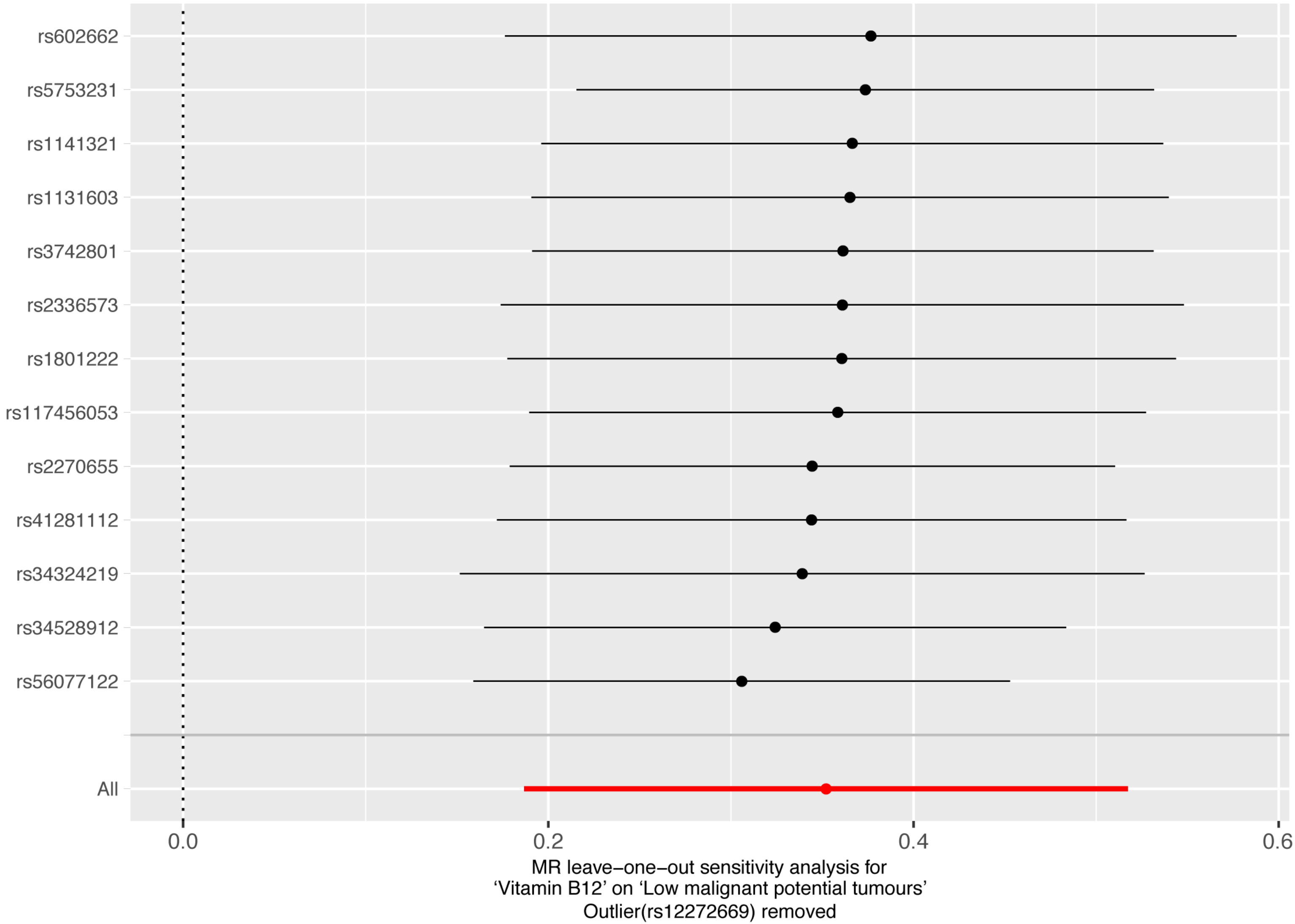

rs602662
rs5753231
rs1141321
rs1131603
rs3742801
rs2336573
rs1801222
rs117456053
rs2270655
rs41281112
rs34324219
rs34528912
rs56077122
All
0.0
0.2
0.4
0.6
MR leave−one−out sensitivity analysis for
'Vitamin B12' on 'Low malignant potential tumours'
Outlier(rs12272669) removed

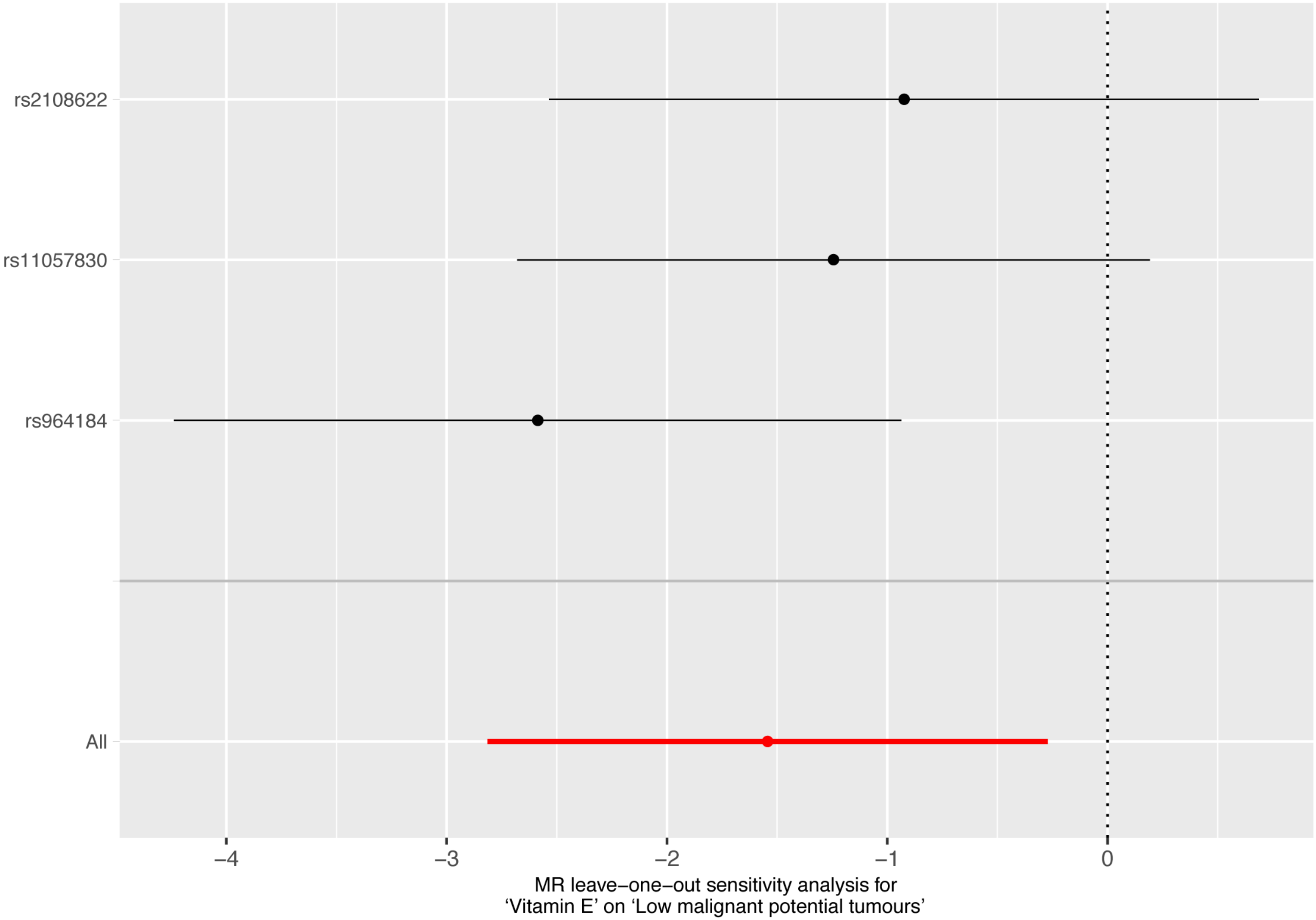
rs2108622
rs11057830
rs964184
All
−4
−3
−2
−1
0
MR leave−one−out sensitivity analysis for
'Vitamin E' on 'Low malignant potential tumours'

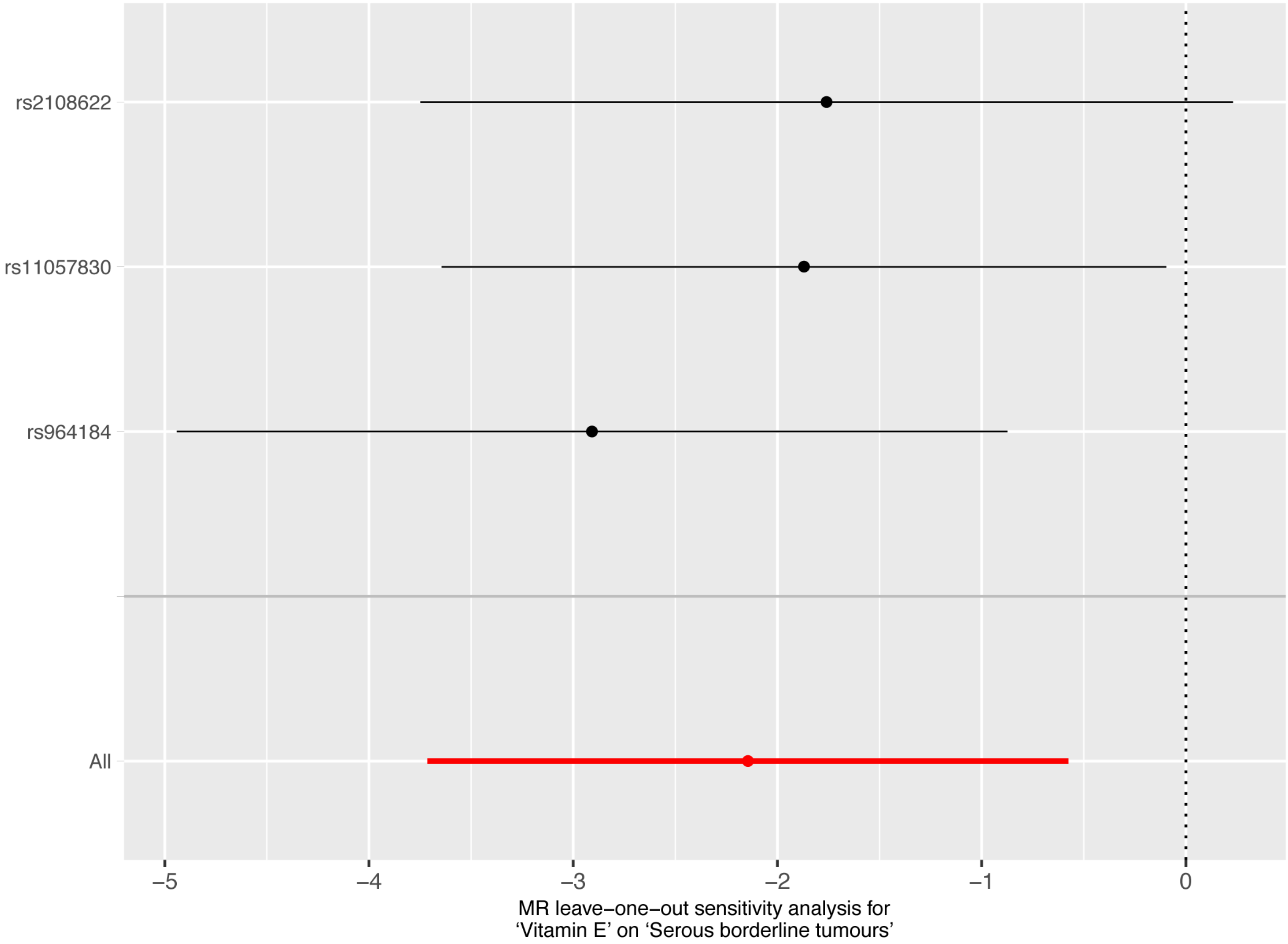
rs2108622
rs11057830
rs964184
All
−5
−4
−3
−2
−1
0
MR leave−one−out sensitivity analysis for
'Vitamin E' on 'Serous borderline tumours'

Table S1. MR analysis estimates for the association of minerals with risk of invasive epithelial ovarian cancer histotypes and low malignant potential tumours

Risk factor	Ovarian cancer outcome	IVW OR (95% CI)	*P*-value	Weighted Median OR (95% CI)	*P*-value	MR-Egger regression OR (95% CI)	*P*-value	MR-RAPS OR (95%CI)	*P*-value	MR-Egger intercept	*P*-value
**Fe**											
	IEOC	0.99(0.90-1.09)	0.86	0.99(0.88-1.11)	0.86	0.75(0.50-1.13)	0.40	0.99(0.90-1.09)	0.86	0.06	0.40
	HGSC	1.01(0.91-1.14)	0.81	1.03(0.91-1.17)	0.62	0.83(0.51-1.35)	0.58	1.01(0.90-1.14)	0.81	0.04	0.55
	LGSC	0.81(0.57-1.14)	0.23	0.92(0.59-1.43)	0.71	0.75(0.02-35.17)	0.91	0.76(0.42-1.35)	0.35	0.02	0.97
	Mucinous	0.96(0.72-1.27)	0.78	0.95(0.69-1.32)	0.77	1.19(0.25-5.70)	0.87	0.96(0.72-1.28)	0.78	-0.05	0.83
	Endometrioid	0.96(0.78-1.19)	0.74	0.93(0.70-1.22)	0.59	0.40(0.13-1.24)	0.36	0.89(0.65-1.22)	0.48	0.19	0.36
	Clear cell	1.14(0.86-1.52)	0.35	1.12(0.79-1.59)	0.53	3.15(0.97-10.21)	0.31	1.15(0.84-1.56)	0.39	-0.22	0.33
	LMP	1.06(0.87-1.30)	0.57	1.12(0.88-1.42)	0.36	0.65(0.27-1.54)	0.51	1.06(0.86-1.31)	0.58	0.11	0.46
	SBT	1.16(0.90-1.49)	0.25	1.20(0.87-1.65)	0.27	0.59(0.20-1.73)	0.51	1.16(0.90-1.50)	0.26	0.14	0.43
	MBT	0.94(0.68-1.29)	0.69	0.92(0.65-1.31)	0.66	0.72(0.19-2.77)	0.72	0.94(0.68-1.30)	0.70	0.06	0.76
**Cu***											
	IEOC	1.02(0.93-1.11)	0.68	-	-	-	-	1.02(0.88-1.18)	0.81	-	-
	HGSC	1.04(0.94-1.16)	0.46	-	-	-	-	1.04(0.86-1.25)	0.69	-	-
	LGSC	1.06(0.77-1.46)	0.73	-	-	-	-	1.06(0.76-1.47)	0.74	-	-
	Mucinous	1.08(0.83-1.41)	0.56	-	-	-	-	1.08(0.82-1.42)	0.57	-	-
	Endometrioid	1.03(0.85-1.25)	0.73	-	-	-	-	1.03(0.85-1.26)	0.74	-	-
	Clear cell	0.95(0.72-1.24)	0.70	-	-	-	-	0.94(0.58-1.51)	0.79	-	-
	LMP	1.11(0.91-1.33)	0.30	-	-	-	-	1.11(0.91-1.35)	0.32	-	-
	SBT	1.15(0.91-1.45)	0.23	-	-	-	-	1.16(0.84-1.58)	0.36	-	-
	MBT	1.00(0.75-1.35)	0.98	-	-	-	-	1.00(0.74-1.36)	0.98	-	-
**Zn**											
	IEOC	0.98(0.91-1.06)	0.60	0.97(0.88-1.06)	0.52	0.87(0.60-1.27)	0.61	0.98(0.90-1.06)	0.61	0.03	0.65
	HGSC	0.96(0.88-1.05)	0.36	0.99(0.89-1.10)	0.91	1.02(0.56-1.86)	0.95	0.96(0.87-1.05)	0.38	-0.01	0.86
	LGSC	0.91(0.69-1.21)	0.52	0.97(0.69-1.36)	0.87	0.98(0.25-3.91)	0.98	0.91(0.68-1.22)	0.53	-0.02	0.93
	Mucinous	0.82(0.65-1.04)	0.10	0.76(0.58-1.01)	0.06	0.78(0.14-4.23)	0.82	0.82(0.64-1.05)	0.12	0.01	0.96
	Endometrioid	0.97(0.82-1.15)	0.75	0.95(0.78-1.17)	0.63	0.47(0.21-1.06)	0.32	0.97(0.78-1.19)	0.74	0.17	0.32
	Clear cell	1.08(0.85-1.37)	0.55	1.04(0.80-1.36)	0.76	1.13(0.36-3.58)	0.87	1.08(0.84-1.38)	0.56	-0.01	0.95
	LMP	0.91(0.77-1.08)	0.27	0.91(0.76-1.09)	0.31	0.70(0.31-1.57)	0.55	0.91(0.77-1.08)	0.28	0.06	0.63
	SBT	0.87(0.71-1.06)	0.17	0.86(0.68-1.08)	0.19	0.78(0.29-2.08)	0.70	0.87(0.70-1.07)	0.19	0.03	0.86

	MBT	1.00(0.77-1.31)	0.97	1.02(0.77-1.34)	0.92	0.60(0.17-2.11)	0.57	1.00(0.77-1.32)	0.97	0.12	0.56
**Ca**											
	IEOC	1.24(0.78-1.98)	0.36	1.32(0.84-2.07)	0.23	1.51(0.62-3.71)	0.41	1.30(0.82-2.07)	0.27	-0.01	0.63
	HGSC	1.41(0.74-2.67)	0.30	1.45(0.84-2.53)	0.18	1.73(0.49-6.07)	0.43	1.48(0.77-2.81)	0.24	-0.01	0.71
	LGSC	1.2(0.12-11.55)	0.88	0.52(0.08-3.30)	0.49	0.16(2.88e-3-8.85)	0.41	0.88(0.11-7.06)	0.90	0.07	0.29
	Mucinous	0.82(0.28-2.40)	0.72	0.99(0.23-4.27)	0.99	1.04(0.11-9.80)	0.97	0.90(0.25-3.20)	0.87	-0.01	0.81
	Endometrioid	0.54(0.23-1.28)	0.16	0.62(0.23-1.72)	0.36	0.71(0.13-3.90)	0.71	0.54(0.21-1.39)	0.20	-0.01	0.72
	Clear cell	1.33(0.49-3.65)	0.58	1.67(0.39-7.11)	0.49	2.90(0.32-26.76)	0.39	1.34(0.37-4.77)	0.66	-0.03	0.45
	LMP	1.90(0.80-4.48)	0.14	1.43(0.54-3.75)	0.47	0.94(0.20-4.50)	0.94	1.91(0.77-4.74)	0.16	0.02	0.34
	SBT	1.36(0.51-3.65)	0.54	0.78(0.24-2.50)	0.67	0.43(0.06-2.96)	0.43	1.36(0.45-4.14)	0.59	0.04	0.22
	MBT	3.29(1.14-9.53)	0.03	2.71(0.58-12.71)	0.21	3.12(0.28-35.05)	0.40	3.20(0.80-12.79)	0.10	0.00	0.96
**Mg**											
	IEOC	0.14(0.03-0.70)	0.02	0.19(0.02-1.67)	0.13	3.33(0.02-5.32e2)	0.67	0.12(0.02-0.73)	0.02	-0.02	0.26
	HGSC	0.49(0.05-4.93)	0.55	1.17(0.08-17.07)	0.91	1.15e2(0.28-4.76e4)	0.20	0.49(0.04-5.98)	0.57	-0.04	0.13
	LGSC	0.01(3.27e-5-5.26)	0.16	0.01(3.78e-6-8.52)	0.17	5.13e-5(5.69e-13-4.63e3)	0.35	0.01(2.57e-5-6.32)	0.17	0.04	0.56
	Mucinous	2.96(0.02-5.54e2)	0.68	3.46(0.01-1.69e3)	0.69	8.96e-4(1.28e-10-6.26e3)	0.43	3.01(0.02-5.51e2)	0.68	0.06	0.35
	Endometrioid	6.46e-4(1.77e-5-0.02)	6.32e-5	5.57e-4(6.38e-6-0.05)	1.01e-3	8.77e-5(1.46e-9-5.25)	0.17	6.28e-4（1.41e-5-0.03）	1.42e-4	0.01	0.73
	Clear cell	1.05E-2(2.48e-5-4.44)	0.14	0.01(1.48e-1-2.44)	0.22	9.74e2(4.28e-5-2.21e10)	0.47	4.15e-3((7.75e-6-2.22)	0.09	-0.09	0.23
	LMP	0.11(3.1e-3-3.77)	0.22	0.31(1.01e-3-96.68)	0.69	31.24(1.02e-6-9.55e8)	0.72	0.03(1.42e-4-6.35)	0.20	-0.04	0.53
	SBT	1.08(0.01-85.42)	0.97	1.33(3.02e-3-589.26)	0.93	6.39e2(5.51e-5-7.41e9)	0.48	0.39(2.56e-3-60.29)	0.72	-0.05	0.46
	MBT	2e-3(1.2e-6-3.36)	0.10	4.33e-3(2.14e-6-8.79)	0.16	(2.14e-12-1.15e10)	0.89	1.33e-3(4.61e-7-3.85)	0.10	-0.03	0.74
**P**											
	IEOC	1.16(0.88-1.53)	0.28	1.06(0.68-1.65)	0.80	2.25(0.36-14.22)	0.48	1.16(0.79-1.71)	0.44	-0.03	0.55
	HGSC	1.40(1.05-1.85)	0.02	1.40(0.81-2.41)	0.23	1.39(0.16-12.50)	0.80	1.40(0.89-2.21)	0.15	0.00	1.00
	LGSC	0.99(0.22-4.40)	0.99	0.93(0.16-5.34)	0.94	0.09(1.73e-5-449.06)	0.63	1.04(0.20-5.38)	0.96	0.11	0.63
	Mucinous	1.65(0.75-3.60)	0.21	1.30(0.35-4.82)	0.70	29.99(0.13-7.15e3)	0.35	1.65(0.53-5.20)	0.39	-0.13	0.40
	Endometrioid	0.89(0.42-1.88)	0.76	0.93(0.33-2.61)	0.89	2.93(0.04-2.1e2)	0.67	0.89(0.38-2.04)	0.78	-0.06	0.63
	Clear cell§	1.04(0.72-1.50)	0.83	1.02(0.19-5.42)	0.98	1.58(0.01-4.32e2)	0.90	1.04(0.24-4.52)	0.96	-0.02	0.90
	LMP	0.83(0.40-1.73)	0.62	0.81(0.32-2.07)	0.66	3.58(0.07-1.96e2)	0.60	0.83(0.36-1.90)	0.65	-0.07	0.54
	SBT	1.08(0.81-1.45)	0.59	1.07(0.35-3.29)	0.91	3.76(0.03-4.91e2)	0.65	1.08(0.40-2.95)	0.87	-0.06	0.66
	MBT	0.57(0.09-3.75)	0.56	0.67(0.14-3.12)	0.61	5.32(8.43e-5-3.37e5)	0.79	0.66(0.18-2.48)	0.54	-0.10	0.72
**Se***											
	IEOC	1.00(0.91-1.09)	0.93	-	-	-	-	1.00(0.91-1.09)	0.93	-	-
	HGSC	0.92(0.83-1.02)	0.13	-	-	-	-	0.92(0.83-1.03)	0.15	-	-
	LGSC	1.32(0.97-1.82)	0.08	-	-	-	-	1.33(0.96-1.84)	0.09	-	-

Mucinous	1.04(0.80-1.35)	0.78	-	-	-	-	1.04(0.79-1.36)	0.79	-	-
Endometrioid	1.18(0.98-1.42)	0.09	-	-	-	-	1.18(0.97-1.43)	0.10	-	-
Clear cell	1.25(0.96-1.63)	0.10	-	-	-	-	1.25(0.95-1.64)	0.11	-	-
LMP	0.90(0.75-1.09)	0.28	-	-	-	-	0.90(0.74-1.09)	0.30	-	-
SBT	0.97(0.77-1.22)	0.77	-	-	-	-	0.97(0.76-1.22)	0.78	-	-
MBT	0.85(0.63-1.14)	0.28	-	-	-	-	0.85(0.63-1.15)	0.29	-	-

*Missing data due to not enough instrumental variables.

§MR-Pleiotropy Residual Sum and Outlier (PRESSO) IV outlier detected: rs1697421.

IVW: Inverse-variance weighted; OR, odds ratio; IEOC: invasive epithelial ovarian cancer; HGSC: High grade serous carcinoma; LGSC: Low grade serous carcinoma; LMP: Low malignant potential tumours; SBT: Serous borderline tumors; MBT: Mucinous borderline tumours.

Table S2. MR analysis estimates for the association of vitamins with risk of invasive epithelial ovarian cancer histotypes and low malignant potential tumours

Risk factor	Ovarian cancer outcome	IVW OR (95% CI)	*P*-value	Weighted Median OR (95% CI)	*P*-value	MR-Egger regression OR (95% CI)	*P*-value	MR-RAPS OR (95%CI)	*P*-value	MR-Egger intercept	*P*-value
**Vitamin A***											
	IEOC	0.82(0.44-1.56)	0.55	-	-	-	-	0.81(0.25-2.68)	0.73	-	-
	HGSC	1.00(0.47-2.13)	1.00	-	-	-	-	0.98(0.17-5.61)	0.98	-	-
	LGSC	1.29(0.13-12.9)	0.83	-	-	-	-	1.29(0.12-13.75)	0.83	-	-
	Mucinous	0.18(0.03-1.21)	0.08	-	-	-	-	0.18(0.02-1.31)	0.09	-	-
	Endometrioid	0.45(0.11-1.78)	0.25	-	-	-	-	0.44(0.10-2.01)	0.29	-	-
	Clear cell	0.40(0.06-2.75)	0.35	-	-	-	-	0.40(0.05-2.92)	0.37	-	-
	LMP	0.65(0.17-2.51)	0.53	-	-	-	-	0.62(0.03-13.05)	0.76	-	-
	SBT	0.72(0.14-3.81)	0.70	-	-	-	-	0.71(0.07-6.93)	0.77	-	-
	MBT	0.64(0.08-5.26)	0.67	-	-	-	-	0.6(0.01-38.10)	0.81	-	-
**β-carotene**											
	IEOC	1.04(1.003-1.09)	0.03	1.04(0.92-1.17)	0.51	1.03(0.49-2.15)	0.95	1.04(0.95-1.15)	0.39	0.002	0.97
	HGSC	1.04(0.97-1.11)	0.3	1.01(0.89-1.16)	0.85	1.11(0.46-2.67)	0.84	1.04(0.92-1.17)	0.55	-0.01	0.89
	LGSC	0.76(0.67-0.86)	1.85e-5	0.77(0.52-1.13)	0.18	1.12(0.08-16.27)	0.94	0.76(0.53-1.09)	0.13	-0.05	0.80
	Mucinous	1.21(1.07-1.37)	2.95e-3	1.29(0.93-1.78)	0.13	0.96(0.11-8.66)	0.98	1.21(0.90-1.63)	0.21	0.03	0.86
	Endometrioid	1.10(1.05-1.15)	4.02e-5	1.12(0.88-1.42)	0.36	1.39(0.28-6.84)	0.72	1.10(0.89-1.36)	0.38	-0.03	0.80
	Clear cell	1.08(0.90-1.29)	0.39	1.04(0.74-1.48)	0.81	2.01(0.22-18.65)	0.60	1.08(0.80-1.46)	0.61	-0.09	0.64
	LMP	0.82(0.76-0.90)	1.01e-5	0.82(0.64-1.05)	0.11	0.63(0.13-3.02)	0.62	0.82(0.67-1.02)	0.08	0.04	0.76
	SBT	1.01(0.87-1.16)	0.93	1.03(0.77-1.38)	0.85	0.82(0.12-5.67)	0.86	1.01(0.78-1.31)	0.96	0.03	0.85
	MBT	0.57(0.53-0.61)	3.89e-53	0.58(0.40-0.84)	3.57e-3	0.49(0.04-5.65)	0.62	0.57(0.40-0.80)	1.02e-3	0.02	0.91
**Vitamin B6***											
	IEOC	-	-	-	-	-	-	1.00(0.98-1.02)	0.99	-	-
	HGSC	-	-	-	-	-	-	1.01(0.99-1.04)	0.24	-	-
	LGSC	-	-	-	-	-	-	0.98(0.92-1.05)	0.66	-	-
	Mucinous	-	-	-	-	-	-	1.00(0.95-1.06)	0.91	-	-
	Endometrioid	-	-	-	-	-	-	0.98(0.94-1.02)	0.33	-	-
	Clear cell	-	-	-	-	-	-	0.91(0.85-0.97)	0.005	-	-

LMP	-	-	-	-	-	-	1.00(0.96-1.04)	0.88	-	-
SBT	-	-	-	-	-	-	1.00(0.95-1.05)	0.92	-	-
MBT	-	-	-	-	-	-	1.00(0.94-1.06)	0.98	-	-
**Vitamin B12**										
IEOC	0.99(0.92-1.06)	0.70	0.97(0.88-1.07)	0.60	0.96(0.85-1.09)	0.56	0.97(0.90-1.05)	0.49	0.004	0.65
HGSC	0.94(0.87-1.01)	0.08	0.95(0.85-1.06)	0.32	0.97(0.85-1.10)	0.61	0.93(0.86-1.01)	0.09	-0.01	0.56
LGSC	0.95(0.74-1.23)	0.72	1.03(0.75-1.41)	0.86	0.85(0.56-1.31)	0.48	0.98(0.75-1.29)	0.91	0.02	0.53
Mucinous	1.02(0.86-1.21)	0.82	0.91(0.68-1.21)	0.50	0.85(0.61-1.19)	0.37	1.01(0.82-1.24)	0.95	0.03	0.21
Endometrioid	1.06(0.90-1.26)	0.49	1.05(0.85-1.29)	0.68	0.87(0.68-1.12)	0.31	1.02(0.85-1.21)	0.86	0.04	0.08
Clear cell	1.15(0.89-1.48)	0.30	1.07(0.80-1.44)	0.64	1.04(0.67-1.61)	0.86	1.09(0.85-1.39)	0.50	0.02	0.60
LMP§	1.42(1.21-1.68)	2.99e-5	1.33(1.05-1.69)	0.02	1.57(1.08-2.28)	0.04	1.41(1.17-1.70)	3.2e-4	-0.01	0.57
SBT	1.23(1.00-1.51)	0.05	1.17(0.93-1.48)	0.18	1.23(0.86-1.74)	0.28	1.22(0.99-1.51)	0.06	5.52e-4	0.98
MBT	1.06(0.75-1.51)	0.73	1.02(0.67-1.55)	0.93	0.66(0.40-1.10)	0.14	1.33(1.03-1.71)	0.03	21.18	0.05
**Vitamin E**										
IEOC	0.84(0.47-1.52)	0.57	0.84(0.34-2.08)	0.71	0.73(0.01-59.18)	0.91	0.84(0.46-1.55)	0.58	0.00	0.96
HGSC	1.01(0.50-2.04)	0.98	1.08(0.44-2.66)	0.87	3.1(0.02-563.02)	0.74	1.01(0.48-2.15)	0.98	-0.04	0.74
LGSC	1.97(0.23-16.68)	0.54	2.58(0.12-55.52)	0.54	2.87e-5(2.91e-12-2.83e2)	0.42	2.05(0.18-22.88)	0.56	0.37	0.40
Mucinous	1.00(0.17-5.89)	0.99	0.44(0-6.62e3)	0.87	5.88e-4(6.8e-10-5.09e2)	0.48	0.97(0.12-7.71)	0.98	0.25	0.48
Endometrioid	0.94(0.26-3.39)	0.92	0.66(0.09-4.87)	0.68	0.01(7.81e-9-1.70e4)	0.65	0.86(0.16-4.58)	0.86	0.15	0.65
Clear cell	0.18(0.03-1.10)	0.06	0.15(0.01-1.76)	0.13	38.37(5.40e-5-2.72e7)	0.69	0.18(0.02-1.32)	0.09	-0.18	0.58
LMP	0.21(0.06-0.76)	0.02	0.21(0.04-1.20)	0.08	2.17e3(0.18-2.6e7)	0.35	0.2(0.03-1.22)	0.08	-0.31	0.30
SBT	0.12(0.02-0.56)	0.01	0.12(0.01-1.06)	0.06	1.03e2(9.44e-4-1.14e7)	0.58	0.11(0.02-0.72)	0.02	-0.23	0.45
MBT	0.62(0.09-4.52)	0.64	0.32(0.03-4.02)	0.38	4.52e4(0.02-9.73e10)	0.39	0.60(0.06-5.68)	0.66	-0.37	0.37
**Folate**										
IEOC	0.94(0.77-1.16)	0.59	0.84(0.66-1.08)	0.17	1.18(0.10-13.38)	0.92	0.95(0.75-1.19)	0.65	-0.02	0.89
HGSC	0.99(0.77-1.26)	0.92	0.95(0.70-1.29)	0.75	1.85(0.07-51.48)	0.78	1.00(0.72-1.39)	0.99	-0.06	0.77
LGSC	0.85(0.41-1.79)	0.67	0.69(0.29-1.62)	0.39	1.12(2.75e-3-452.52)	0.98	0.85(0.40-1.83)	0.68	-0.03	0.94
Mucinous	1.06(0.58-1.96)	0.84	0.90(0.47-1.72)	0.74	0.02(2.59e-4-1.56)	0.33	1.02(0.54-1.92)	0.95	0.37	0.32
Endometrioid	0.83(0.54-1.29)	0.42	0.84(0.52-1.35)	0.47	2.88(0.11-73.79)	0.64	0.83(0.53-1.31)	0.43	-0.12	0.59
Clear cell	1.17(0.63-2.17)	0.63	1.22(0.62-2.38)	0.57	6.58(0.07-6.14e2)	0.57	1.17(0.62-2.21)	0.64	-0.16	0.59
LMP	0.86(0.56-1.33)	0.49	0.87(0.53-1.43)	0.59	0.56(0.02-13.95)	0.78	0.86(0.55-1.34)	0.50	0.04	0.83
SBT	0.97(0.57-1.66)	0.91	0.98(0.55-1.76)	0.95	0.11(6.20e-4-17.92)	0.55	1.00(0.53-1.89)	1.00	0.21	0.55
MBT	0.72(0.36-1.42)	0.34	0.79(0.38-1.65)	0.53	5.9(0.04-944.83)	0.62	0.72(0.35-1.45)	0.35	-0.2	0.56

*Missing data due to not enough instrumental variables

§MR-Pleiotropy Residual Sum and Outlier (PRESSO) IV outlier detected: rs12272669.
IVW: Inverse-variance weighted; OR, odds ratio; IEOC: invasive epithelial ovarian cancer; HGSC: High grade serous carcinoma; LGSC: Low grade serous carcinoma; LMP: Low malignant potential tumours; SBT: Serous borderline tumours; MBT: Mucinous borderline tumours.
